# Preventive Treatments for Psychosis: Umbrella Review (Just the Evidence)

**DOI:** 10.3389/fpsyt.2019.00764

**Published:** 2019-12-11

**Authors:** Paolo Fusar-Poli, Cathy Davies, Marco Solmi, Natascia Brondino, Andrea De Micheli, Magdalena Kotlicka-Antczak, Jae Il Shin, Joaquim Radua

**Affiliations:** ^1^Early Psychosis: Interventions and Clinical-detection (EPIC) Lab, Department of Psychosis Studies, Institute of Psychiatry, Psychology & Neuroscience, King’s College London, London, United Kingdom; ^2^OASIS Service, South London and Maudsley NHS Foundation Trust, London, United Kingdom; ^3^Department of Brain and Behavioral Sciences, University of Pavia, Pavia, Italy; ^4^National Institute for Health Research, Maudsley Biomedical Research Centre, South London and Maudsley NHS Foundation Trust, London, United Kingdom; ^5^Neuroscience Department, Psychiatry Unit, Padua Neuroscience Center, University of Padua, Padua, Italy; ^6^Department of Affective and Psychotic Disorders, Medical University of Lodz, Lodz, Poland; ^7^Department of Pediatrics, Yonsei University College of Medicine, Seoul, South Korea; ^8^Institut d’Investigacions Biomèdiques August Pi i Sunyer (IDIBAPS), CIBERSAM, Barcelona, Spain; ^9^Department of Clinical Neuroscience, Centre for Psychiatric Research and Education, Karolinska Institutet, Stockholm, Sweden

**Keywords:** psychosis, schizophrenia, prevention, treatments, meta-analysis, evidence

## Abstract

**Background:** Indicated primary prevention in young people at Clinical High Risk for Psychosis (CHR-P) is a promising avenue for improving outcomes of one of the most severe mental disorders but their effectiveness has recently been questioned.

**Methods:** Umbrella review. A multi-step independent literature search of Web of Science until January 11, 2019, identified interventional meta-analyses in CHR-P individuals. The individual randomised controlled trials that were analysed by the meta-analyses were extracted. A review of ongoing trials and a simulation of living meta-analysis complemented the analysis.

**Results:** Seven meta-analyses investigating preventive treatments in CHR-P individuals were included. None of them produced pooled effect sizes across psychological, pharmacological, or other types of interventions. The outcomes analysed encompassed risk of psychosis onset, the acceptability of treatments, the severity of attenuated positive/negative psychotic symptoms, depression, symptom-related distress, social functioning, general functioning, and quality of life. These meta-analyses were based on 20 randomised controlled trials: the vast majority defined the prevention of psychosis onset as their primary outcome of interest and only powered to large effect sizes. There was no evidence to favour any preventive intervention over any other (or control condition) for improving any of these clinical outcomes. Caution is required when making clinical recommendations for the prevention of psychosis in individuals at risk.

**Discussion:** Prevention of psychosis from a CHR-P state has been, and should remain, the primary outcome of interventional research, refined and complemented by other clinically meaningful outcomes. Stagnation of knowledge should promote innovative and collaborative research efforts, in line with the progressive and incremental nature of medical knowledge. Advancements will most likely be associated with the development of new experimental therapeutics that are ongoing along with the ability to deconstruct the high heterogeneity within CHR-P populations. This would require the estimation of treatment-specific effect sizes through living individual participant data meta-analyses, controlling risk enrichment during recruitment, statistical power, and embedding precision medicine within youth mental health services that can accommodate sequential prognosis and advanced trial designs.

**Conclusions:** The evidence-based challenges and proposed solutions addressed by this umbrella review can inform the next generation of research into preventive treatments for psychosis.

## Introduction

Prevention of psychosis is a promising avenue for improving outcomes of one of the most severe mental disorders (1). It entails three stepped core components: efficient detection of individuals at risk, an accurate prognosis of outcomes, and an effective preventive treatment that can impact the course of the disorder (2). These activities are usually managed by specialised clinical services. The first rate-limiting step is the detection of children, adolescents and young adults aged 8–40 (3) (more frequently 14–35) (4) who may be at risk of developing psychosis. Their detection is based on recruitment campaigns (5) that filter individuals who have accumulated several risk factors (6) for the development of psychosis, thus enriching the level of risk. The second step is the clinical assessment of these individuals and the formulation of a prognosis (7–9). Individuals meeting psychometric intake criteria for a Clinical High Risk state for Psychosis (CHR-P (10)) are functionally impaired (11) and have a 20% risk of developing psychosis at 2 years (12)] [but not an increased risk of developing other non-psychotic mental disorders (13, 14)]. The third, final step is the provision of an effective intervention, which can impact the clinical outcomes of CHR-P individuals. Evidence of effective interventions in CHR-P individuals—also termed as primary indicated interventions (15)—will be the focus of the current manuscript.

**Figure 1:**
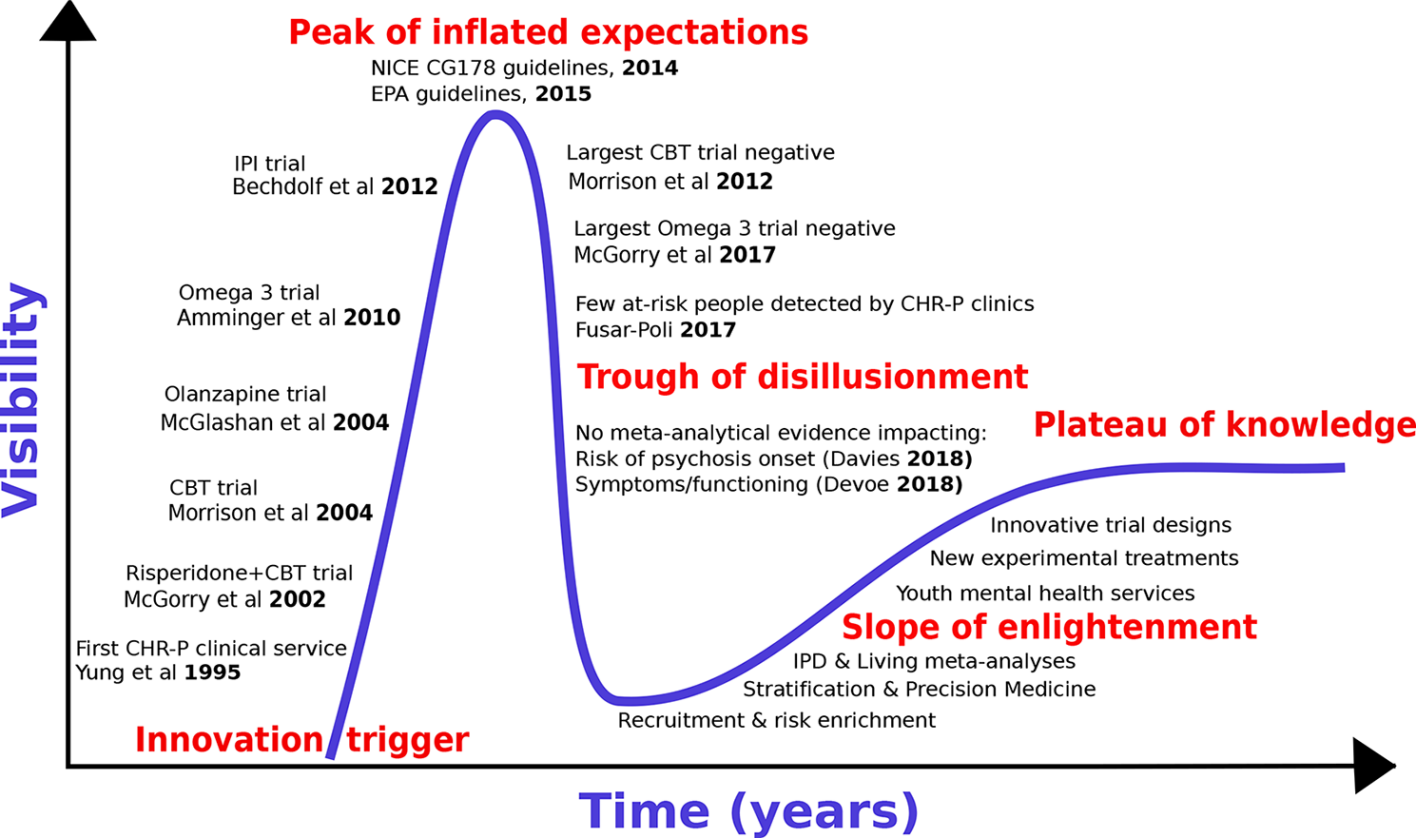
The hype cycle of preventive treatments for psychosis.

We first review the historical development of preventive treatments for psychosis by adapting the “Gartner Hype Cycle (16)” model to the CHR-P paradigm [for details, see (17)]. The Hype Cycle has been used to describe the course of knowledge in other areas of medical knowledge [e.g., machine-learning (18)]. The Gartner Hype Cycle describes the course of new technological discoveries, assuming that humans tend to overestimate the impact of a new discovery in the short term, while largely underestimating the same in the long term. The Hype Cycle—illustrated in **Figure 1**—includes five different stages: 1) innovative trigger, 2) peak of inflated expectations, 3) trough of disillusionment, 4) slope of enlightenment, and 5) plateau of productivity or knowledge.

With reference to the CHR-P paradigm, the innovative trigger for the development of preventive treatments for psychosis was the set-up of the first CHR-P clinic (in 1995), followed (in 2002) by the publication of the first randomised controlled trial in CHR-P individuals. Such a trial employed antipsychotic (risperidone) and psychological treatment, and demonstrated that they were effective in reducing the risk of developing a first episode of psychosis in CHR-P samples (19). Over the ensuing years, the innovative trigger led to an explosion of enthusiasm with the development of additional treatments that—it was hoped—could prevent the onset of psychosis from a CHR-P state. Randomised controlled trials involving antipsychotics [olanzapine, 2006 (20), psychological therapies [Cognitive Behavioural Therapy (21), 2004 or Integrated Psychological Intervention (22), 2012] and dietary interventions [Omega-3 (23)] all confirmed some degree of efficacy for reducing the onset of psychosis in CHR-P individuals (**Figure 1**). The peak of inflated expectations and optimism was likely reached through the publication of the National Institute for Health and Care Excellence (NICE) Clinical Guidelines 178 (2014) (24) and the European Psychiatric Association (EPA) guidelines [2015 (25)] These guidelines set the gold standard for the prevention of psychosis in CHR-P individuals in clinical routine. At the same time, these guidelines also represented the starting point of the trough of disillusionment, because they were grounded on “non-conclusive (26)” and “moderate quality (26)” evidence of small magnitude (risk difference −0.07: 95%CIs −0.14 to −0.01) (26). Upon closer inspection, the two guidelines were already reflecting a lack of robust evidence at the time of their release, because they were partially discordant. In fact, while prophylactic treatment with antipsychotics was altogether not recommended by NICE guidelines (24), the EPA allowed their use in the case of severe and progressive symptomatology (24). Over the following years, the trough of disillusionment for effective preventive treatments for psychosis onset received additional corroborating support. Two large randomized controlled trials showed that neither cognitive behavioral therapy [2012 (27)] nor Omega-3 interventions [2017/2018 (28, 29)] are effective in reducing the progression to psychosis from a CHR-P state. Overall, seven new trials involving 992 new CHR-P participants (an increase of more than 50%) have been published since the last meta-analysis that informed the NICE guidelines (30). All of these trials were negative (30, 31). An updated network meta-analysis (2018) (30) incorporating these new randomized controlled trials confirmed the lack of evidence to support specific preventive treatments over each—and every—other for the prevention of psychosis in CHR-P individuals.

The current trough of disillusionment phase has stimulated discussion across the field. On the one hand, some authors have attempted to minimize the clinical relevance of the evidence itself. Specifically, it has been argued that current preventive treatments are actually effective on outcomes other than the prevention of psychosis onset, that recent network meta-analyses should have estimated effects across different types of preventive treatments “pooled together”, and that the primary outcome analysed by these meta-analyses—i.e., transition to psychosis from a CHR-P state—is a “second-order issue” of questionable clinical significance (32). On the other side, some authors have argued that there is now enough evidence to abandon the CHR-P paradigm altogether. The current study explores the area between these two polarities, to recognize the key challenges while at the same time proposing ways to overcome them. To address such an uncertain phase of knowledge, we strictly adopt an evidence-based medicine approach. We will interrogate the published literature by conducting an umbrella review (33), i.e., a review of meta-analyses in the field of preventive interventions for psychosis in CHR-P individuals. Because umbrella reviews are based on meta-analyses previously published, they represent a bird’s-eye view on a determinate topic and reflect one of the highest levels of evidence. First, we will specifically review the evidence that preventive interventions can impact clinical outcomes (including and going beyond the development of psychosis) in CHR-P individuals. We will also review the use of pooled vs treatment-specific effect size estimates. Second, we will systematically review the extent to which prevention of psychosis has been the first or second-order outcome in interventional studies in this population. Third, we will critically interpret the umbrella review findings to inform the next slope of enlightenment stage, which will hopefully deliver a plateau of knowledge in this field.

## Methods

### Search Strategy, Selection Criteria, and Data Extraction for the Systematic Review

A multi-step literature search was performed. First, systematic searches were conducted in the Web of Science (which includes Web of Science Core Collection, BIOSIS Citation Index, KCI—Korean Journal Database, MEDLINE, Russian Science Citation Index, and SciELO Citation Index), until January 11, 2019, with no restrictions on language or publication date. The keywords “psychosis risk” or “clinical high risk” or “at risk mental state” or “prodromal,” “CAARMS” or “SIPS” or “basic symptoms” or “UHR” or “CHR” were used, filtering for the category “review” and “psychiatry” through the Web of Science categories function. Second, we searched the abstracts of retrieved articles to include only those that employed meta-analytical methods. Third, full-text articles identified by this process were then screened and inspected against the inclusion and exclusion criteria.

The literature search, study selection, and data extraction were conducted by two authors independently. During all stages, in the case of disagreement, the consensus was reached through discussion with a third author.

Studies were eligible for inclusion when the following criteria were fulfilled: a) meta-analyses (aggregate or network) in CHR-P individuals [defined according to established international criteria (3)]; b) a clear and primary focus on interventions for this patient population, with no restriction on the design of the primary studies (open-label, controlled/uncontrolled, randomised/unrandomized, naturalistic studies) or on the type of the intervention (medications, psychological, physical, dietary, experimental); c) investigating clinical outcomes in CHR-P individuals. The exclusion criteria were: a) original studies, study protocols, abstracts, systematic reviews without quantitative analyses, and any other non-meta-analytical study; and b) lacking a clear primary focus on interventions for CHR individuals. In the case of two or more articles addressing the same outcome we selected individual participant data meta-analyses over aggregate network meta-analyses over pairwise-meta-analyses, and, in a second step, the most recent study. This was done to respect the hierarchy of the evidence. Research is conducted at different levels: primary research consists of original studies while secondary research comprises qualitative reviews, systematic reviews, and meta-analyses. Within meta-analyses, network meta-analyses offer additional benefits over standard pairwise analyses in that the comparative efficacy of specific interventions can be estimated and ranked, even when two treatments have never been compared directly head-to-head (25). Furthermore, since network meta-analyses can improve the precision of estimates by allowing integration of both direct and indirect treatment effect estimates (26), it is recommended over pairwise meta-analyses by the World Health Organization as a basis for clinical guidelines (27). Therefore, network meta-analyses should be considered the highest level of evidence in CHR-P treatment guidelines (28). Finally, Individual Participant Data meta-analyses should be considered as a higher level of evidence compared to aggregate-data meta-analyses (34), because they can improve the quality of the data, the analyses and thus the reliability of the results. Therefore, they are considered the gold standard. The quality of the meta-analyses identified through the literature search was assessed with the Assessing the Methodological Quality of Systematic Reviews (AMSTAR) tool (35). AMSTAR is a reliable and valid measurement tool to assess the methodological quality of systematic reviews. AMSTAR assess whether the systematic review was based on an “a priori” protocol, if authors run screening and data extraction in duplicates, if they run a comprehensive search, including grey literature, if they provided a complete list of both included and excluded studies (at full-text assessment), if they assessed quality of included studies, and accounted for such quality in conclusions, if used appropriate methods to pool and analyse data, if assessed publication bias, and if disclosed information on potential conflict of interest. AMSTAR score goes from 0 (low quality) to 11 (high quality) (35).

In a subsequent step, we additionally extracted the individual studies that were analysed by the corresponding meta-analysis that was included in the current umbrella review. Specifically, we included only randomised controlled trials with no restriction on study design, being published in the English language and with a sample size of at least 10 participants (36). Finally, to further provide a comprehensive view of current research in this field we systematically searched the clinicaltrials.gov using the keyword “psychosis risk” and filtering for randomised trials that were recruiting or active but not recruiting. The records were then summarized in a descriptive table.

### Simulation of a Living Meta-Analysis

Unfortunately, meta-analyses—even if based on individual participant data—are outdated as soon as new studies on the same topic emerge. Once published, only a minority of meta-analyses are then updated within two years of publication (37). Such an inability to maintain recency may lead to significant inaccuracy in clinical practice which is not updated with evidence-based medicine. For example, by 2 years post-publication, 23% of non-updated meta-analyses will have failed to incorporate new evidence that would substantively change its conclusions (38).

**Figure 2:**
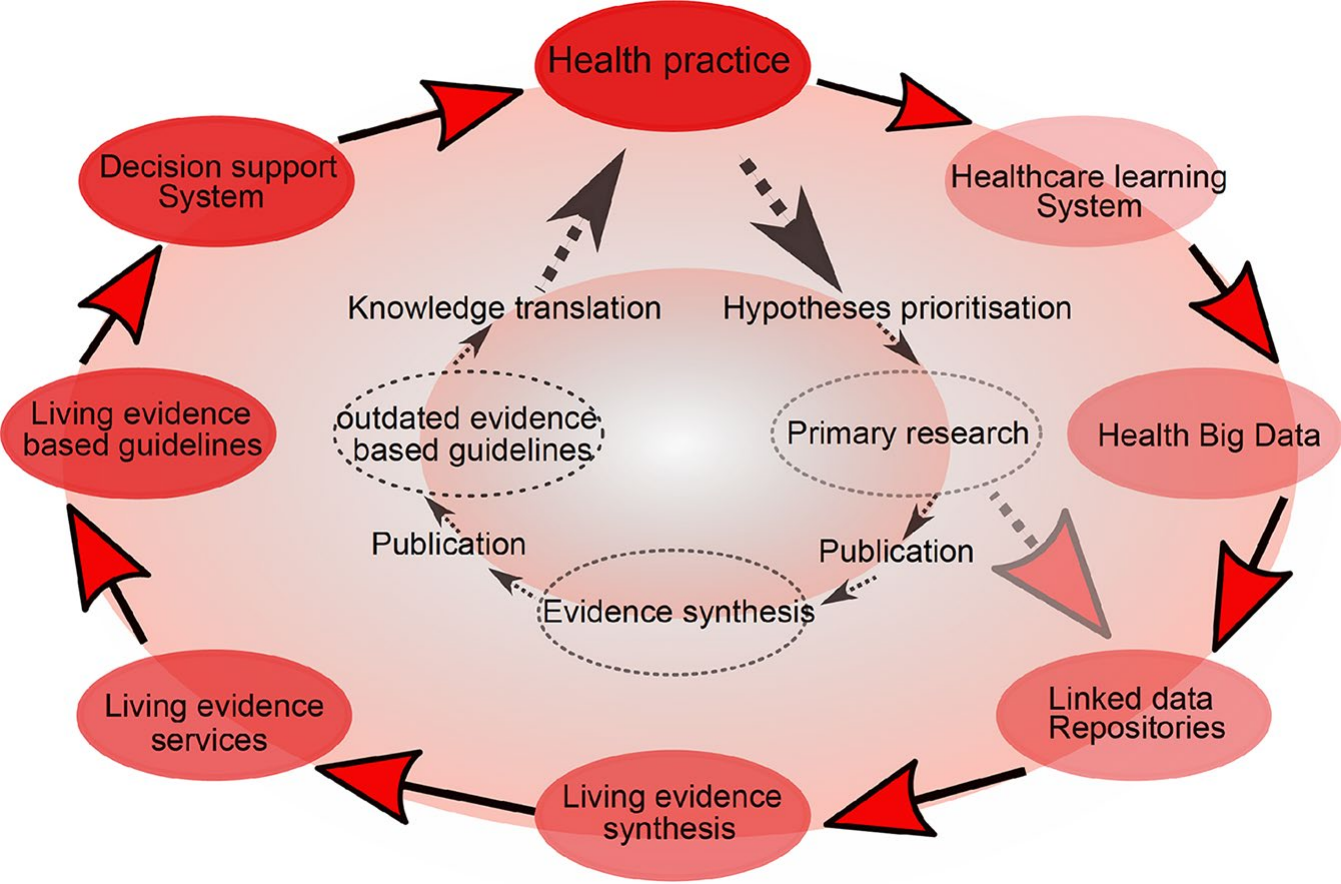
Living meta-analyses. Current (inner circle) and emerging (outer circle) evidence-based health knowledge ecosystems. The current health knowledge ecosystem is characterized by inefficiencies that hamper the flow of knowledge from health practice through primary research, evidence synthesis and guidelines, and finally back to impacts on health practice. The emerging health knowledge ecosystem is characterized by a continuous flow of knowledge between living components, including the growing importance of learning health care systems (a dynamic system which is continuously learning from new data), which together with traditional primary research will populate common data repositories. Living evidence services derived from these repositories, supporting living guidance and decision support systems will close a ‘‘living’’ health knowledge loop. Adapted from (41).

Cumulative meta-analysis, defined as updating a meta-analysis whenever a new eligible RCT becomes available, can be used to address this (39). However, the problem is that the median time taken for a primary study to be incorporated into a meta-analysis ranges from 2.5 to 6.5 years (40). Thus, in 2014, “living systematic reviews” were proposed as a framework for continuously updating meta-analyses (41) (**Figure 2**).

Living meta-analyses are particularly indicated when the question to be addressed is essential to decision-making, when there is some uncertainty of the evidence, when new information is likely to change the findings, and when there is likely to be new evidence (42). All of these conditions apply to the CHR-P field. Crucially, living meta-analyses require bespoke analytical approaches that have been developed for sequential (interim) analyses of randomized clinical trials (43–46). We simulate here a living meta-analysis using the available RCTs. We employed methods which allow reducing the chances of inflating type-I errors (by using the alpha-spending monitoring boundaries (47)) and type II errors (by estimating the required “*a priori* anticipated information size” APIS), i.e., the minimum required sample size to detect an assumed minimal clinically important effect-size—as recommended by experts (48) —with a prespecified statistical power) (49). Furthermore, we used approximate Bayesian approaches to reduce the misestimation of heterogeneity (50). The results are presented in specific plots to facilitate the interpretation of the core findings.

## Results

### Database for the Systematic Review

**Figure 3:**
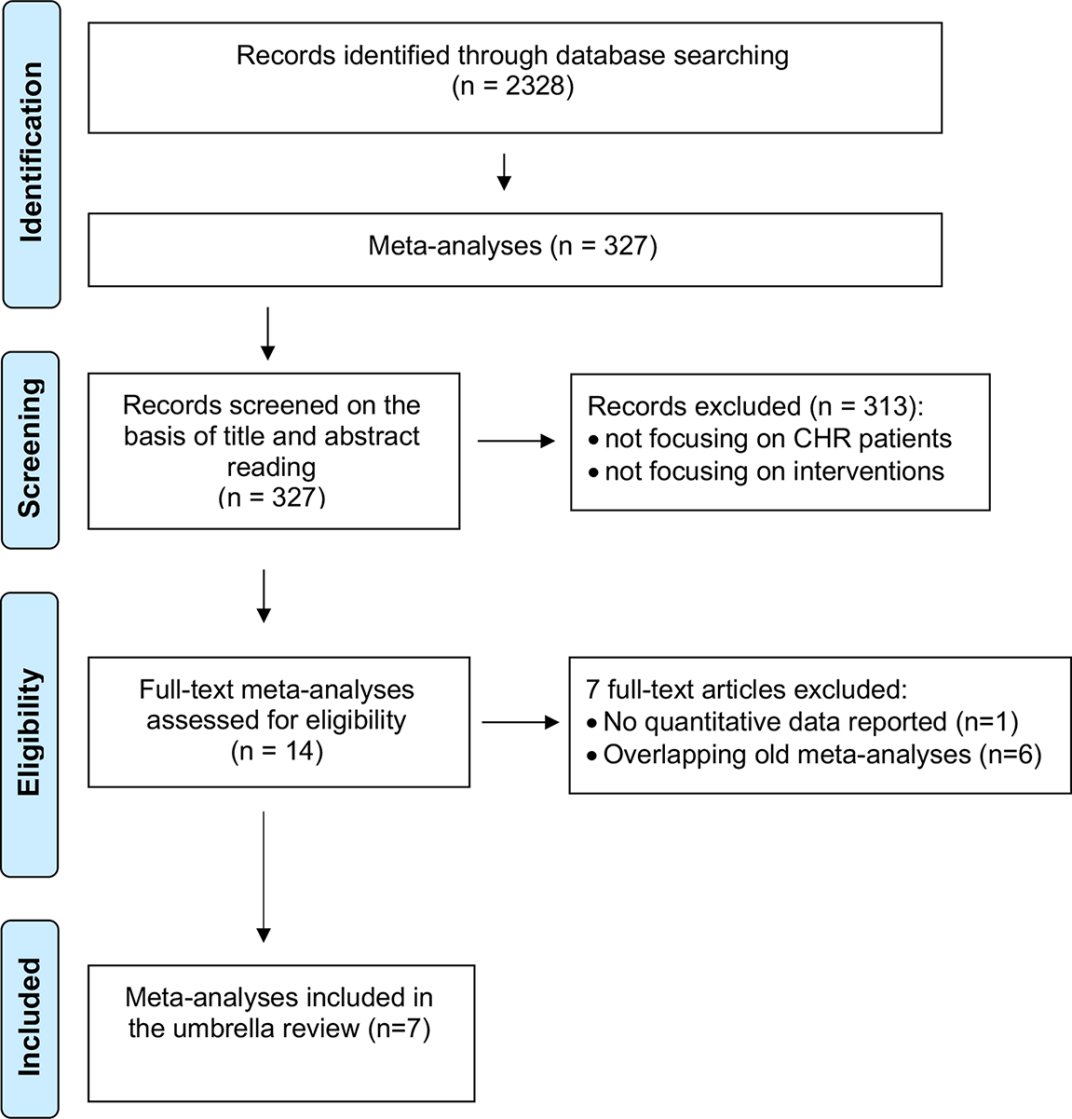
Study identification and selection (PRISMA flowchart).

The initial literature search (**Figure 3**) retrieved 2,328 records, of which only 327 were meta-analyses and were screened on the basis of title and abstract reading.

Most of them were not focusing on CHR-P individuals or interventions, and only 14 full-text aggregate meta-analyses were eventually assessed for eligibility. There were no individual participant data meta-analyses.

One of these 14 meta-analyses was not reporting quantitative data (51) and 6 were overlapping with more recent meta-analyses (25, 52–56): these studies were thus excluded. Since two network meta-analyses addressed the same outcome and were published in the same year (2018) (57, 58), it was not possible to apply our exclusion criteria. Both network meta-analyses were thus included. The final database comprised seven meta-analyses: four aggregate-data network meta-analyses and three aggregate-data pairwise meta-analyses. Additionally, two errata were retrieved, but they were not counted as independent articles (59, 60). The full list of meta-analyses is listed in **Table 1**.

**Table 1:** Efficacy of treatments for CHR-P individuals. Overview of the most recent meta-analyses per clinical outcome (up to January 11^th^, 2019).

Outcome	Author	year	Type of evidence	N of studies (max n of CHR individuals)^a^	AMSTAR rating	Finding
Transition to psychosis	Davies et al. (**30**)	2018	Aggregate Network Meta-Analysis (RCTs)	16 (2, 035)	10/11	Lack of evidence to favor specific treatments
Acceptability	Davies et al. (**30**)	2018	Aggregate Network Meta-Analysis (RCTs)	14 (1, 848)	10/11	Lack of evidence to favor specific treatments
Severity of attenuated positive psychotic symptoms	Davies et al. (**61**)	2018	Aggregate Network Meta-Analysis (RCTs)	14 (1, 707)	10/11	Lack of evidence to favor specific treatments
	Devoe et al. (**58**)	2018	Aggregate Network Meta-Analysis (RCTs)	12 (1, 457)^b^	10/11	Lack of evidence to favor specific treatments
Severity of attenuated negative psychotic symptoms	Devoe et al. (**60**, **62**)	2018	Aggregate Network Meta-Analysis (RCTs)	14 (1, 467)^c^	10/11	Lack of evidence to favor specific treatments
Depression	Stafford et al. (**26**, **59**)	2013	Aggregate Pairwise Meta-Analysis (RCTs)	5 (714)	9/11	No significant treatment effects at any time point
Symptom-related distress	Hutton et al. (**63**)	2014	Aggregate Pairwise Meta-Analysis (RCTs)	Unclear	9/11	No significant treatment effects
Social functioning	Devoe et al. (**64**)	2018	Aggregate Pairwise Meta-Analysis (RCTs)	9 (1, 040)	10/11	No treatment significantly improved social functioning
Functioning	Schmidt et al. (**25**)	2015	Aggregate Pairwise Meta-Analysis (RCTs)	9 (869)	8/11	No significant treatment effects
Quality of life	Hutton et al. (**63**)	2014	Aggregate Pairwise Meta-Analysis (RCTs)	Unclear	9/11	No significant treatment effects

a sample sizes are based on the total sample sizes reported in the meta-analysis minus the sample size of any studies that were not included in their actual meta-analytic computations.

b sample size of Ising et al. (**65**) and the non-randomized arm of McGorry et al. (**66**) (N = 78) not included.

c sample size computed by summing study sample sizes from **Table 1** in Devoe et al. The non-randomized arm of McGorry et al. (**66**) (N = 78) was not included.

### Effect of Preventive Treatments on Clinical Outcomes in CHR Individuals

#### Prevention of Psychosis Onset

An aggregate-data network meta-analysis published in 2018 showed a lack of evidence to favor specific preventive treatments compared to each—and every—other for the prevention of psychosis onset from a CHR state (67). These comparisons also included needs-based interventions or placebo. Some authors speculated that these findings would have changed if the Neurapro trial (28), which was classified as Omega-3 + needs-based intervention vs needs-based intervention [in line with other independent network meta-analyses (58, 62)] was classified as Omega-3 + Cognitive Behavioral Therapy (Cognitive Behavioral Case Management) + needs-based interventions vs cognitive behavioral therapy + needs based interventions (32). As already mentioned in the main paper (30), sensitivity analyses testing different definitions of the nodes did not change the results. For comprehensiveness, we further append in **Figure S1** the forest plot of the network meta-analysis when the Neurapro trial was coded as Omega 3 + Cognitive Behavioral Therapy (Cognitive Behavioral Case Management) + needs-based interventions vs cognitive behavioral therapy + needs-based interventions. The results remained unchanged.

#### Acceptability of Treatments

An aggregate-data network meta-analysis published in 2018 showed a lack of evidence to favor any specific preventive treatment compared to every other (including also needs-based interventions or placebo) with respect to acceptability (defined as treatment drop-outs for any reason) (67).

#### Symptoms

Two independent aggregate-data network meta-analyses published in 2018 found lack of evidence to favor specific preventive treatments compared to each (and every) other for improving attenuated positive psychotic symptoms (58, 61).

Another aggregate-data network meta-analysis published in 2018 demonstrated no evidence to favor specific preventive treatments compared to each (and every) other for the improvement of attenuated negative psychotic symptoms (62). Some authors speculated that this meta-analysis showed “trend-level” benefits for N-methyl-D-aspartate receptor (NMDAR) modulators compared to placebo (32). However, the 95%CI of the Standardized Mean Difference included the null hypothesis (from −1.09 to 0.02) (62). The authors of the network meta-analysis in a following erratum clarified that neither efficacy nor effectiveness was statistically confirmed for any of the examined treatments and that “the abstract and text contained a misstatement regarding effectiveness” (60).

An aggregate-data pairwise meta-analysis published in 2013 indicated that preventive treatments have no impact on depression in CHR-P individuals (26).

A further aggregate-data pairwise meta-analysis published in 2014 found that preventive treatments have no impact on symptom-related distress in CHR-P individuals (63).

#### Functioning

An aggregate-data pairwise meta-analysis published in 2018 found no effect of available preventive treatments for the improvement of social functioning in CHR-P individuals (64). An aggregate-data pairwise meta-analysis published in 2014 found no effect of preventive treatments on a broader level of functioning (63).

#### Quality of Life

The most recent meta-analysis to explore the impact of preventive treatments on quality of life in CHR-P individuals was published in 2014. There were no significant treatment effects reported (63).

### Pooled vs Specific-Treatment Effect Sizes

None of the included meta-analyses estimated overall effect size across different categories of treatments (i.e. medications, psychological, dietary) pooled together.

### Primary Outcomes Investigated by Randomized Controlled Trials in CHR-P Individuals

As indicated in the methods, we further extracted details of the randomized controlled trials that were analyzed by the meta-analyses included in the current umbrella review. A total of 20 trials were retrieved (**Table 2**). The treatments tested were:

- needs-based interventions;
- omega-3 + needs-based interventions;
- ziprasidone + needs-based interventions;
- olanzapine + needs-based interventions;
- aripiprazole + needs-based interventions;
- amisulpride + needs-based interventions;
- Integrated Psychological Interventions;
- family therapy + needs-based interventions;
- D-serine + needs-based interventions;
- cognitive behavioral therapy, French & Morrison protocol + needs-based interventions;
- cognitive behavioral therapy, French & Morrison protocol + risperidone + needs-based interventions;
- cognitive behavioral therapy, van der Gaag protocol + cognitive behavioral therapy, French & Morrison protocol + needs-based interventions;
- cognitive remediation therapy;
- computer games.

**Table 2:** Primary outcome with rationale as declared in the randomized controlled trials of treatments for CHR-P individuals.

Author	Primary outcome	Rationale supporting the primary outcome
Addington et al. (68)	Prevention psychosis	Psychological interventions might be expected to be promising in the pre-psychotic period when the symptoms are less severe and also less specific
Amminger et al. (23)	Prevention psychosis	Intervention in at-risk individuals holds the promise of even better outcomes, with the potential to prevent full blown psychotic disorders.
Bechdolf et al. (22)	Prevention psychosis	Prevention efforts in individuals at imminent risk of schizophrenia can reduce or prevent the devastating effects of the disorder
Bechdolf et al. (69)	Prevention psychosis	Effective interventions for CHR-P individuals are needed in order to reduce or prevent the devastating effects of the disorder
Cadenhead et al. (70)	Prevention psychosis	Replication study testing the efficacy of dietary interventions as defined by Amminger et al. (23)
Choi et al. (71)	Social functioning	Providing cognitive remediation during a putative prodromal stage may improve social functioning and have some value in reducing the risk of psychosis onset
Kantrowitz et al. (72)	Reduction of attenuated negative psychotic symptoms	Negative symptoms and cognitive deficits frequently persist and contribute substantially to impaired functional outcome.
Loewy et al. (73)	Cognitive functioning	The variability in outcomes for CHR-P patients requires treatments that offer the prospect of high benefit and low risk
McGlashan et al. (20)	Prevention psychosis	The chronicity of schizophrenia determines the primary rationale for studies of early intervention for this disorder
McGorry et al. (19)	Prevention psychosis	Progression to psychosis is neither inevitable nor predetermined and it may be possible to delay the onset of psychosis
McGorry et al. (28, 29)	Prevention psychosis	Treatment strategies should relieve distress, improve functioning, and reduce the risk for progression to a psychotic illness
Miklowitz et al. (74)	Reduction of attenuated positive psychotic symptoms	Intervention during the high-risk period may reduce subthreshold psychotic symptoms, enhance social and role functioning, and, over the long term, prevent or delay conversion to episodes of psychosis
Morrison et al. (21)	Prevention psychosis	Specific pharmacotherapy and psychotherapy may be associated with a reduction in progression to psychosis in CHR-P people
Morrison et al. (27)	Prevention psychosis	Effective interventions to prevent or delay this transition are needed because of the significant personal, social, and financial costs associated with the development of psychosis
Piskulic et al. (75)	Cognitive functioning	Given that deficits in cognition are related to poor functional outcome in CHR-P, cognition is a good treatment target
Ruhrmann et al. (76)	Reduction of attenuated positive psychotic symptoms	Attenuated psychotic symptoms are the most important indicators of imminent risk; their disappearance may be associated with lower rates of transition to psychosis
Stain et al. (77)	Prevention psychosis	The CHR-P criteria provide an important opportunity for early intervention in preventing or delaying the onset of psychosis and reducing the social and economic burden associated with long-term mental health problems
van der Gaag et al. (65, 78)	Prevention psychosis	Postponement or prevention of the transition to frank psychosis is the main goal: early detection is of little use without an effective intervention.
Woods et al. (**79**, 80)	Prevention psychosis	To investigate the safety and efficacy of ziprasidone in delaying or preventing conversion to psychosis among individuals meeting CHR-P criteria
Yung et al. (81); McGorry et al. (66)	Prevention psychosis	Cognitive therapy and/or low-dose antipsychotic administered during the prodromal phase of schizophrenia may prevent or delay the onset of full-blown illness.

The primary outcome investigated by these trials as well as the rationale provided to support it are reported in **Table 2**. The vast majority (14/20, 70%) of randomized controlled trials defined the prevention of psychosis onset from a CHR-P state as their primary outcome. Three studies defined as primary outcome the reduction of attenuated psychotic symptoms (72, 74, 76), two studies the improvement of cognitive functioning (73, 75), and one study the improvement of social functioning (71). The AMSTAR scoring for each meta-analysis included is reported in **Table 2**.

### Ongoing Clinical Trials Identified in CHR-P Individuals

Ongoing experimental randomized trials listed in clinicaltrials.gov database that are currently recruiting CHR-P patient are summarized in **Table 3**. Other experimental treatments that are not yet listed in the clinicaltrials.gov database are presented below.

**Table 3:** Ongoing trials in CHR-P individuals.

Trial title; ClinicalTrials.gov Identifier	Population (age); instrument	Trial arms	Allocation and masking	Duration	Sample size	Primary outcome
The Role of Antidepressants or Antipsychotics in Preventing Psychosis: Fluoxetine vs Aripiprazole Comparative Trial (FACT). NCT02357849	CHR-P (12-25); SIPS	1. Fluoxetine; 2. aripiprazole	Randomized (participant, care provider, investigator outcomes assessor)	24 weeks	48	Time to either all-cause-discontinuation or need to add another psychotropic agent
Multimodal Prevention of Psychosis - Investigating Efficacy of N-Acetylcysteine and Psychotherapy in CHR-Patients (ESPRIT-B1). NCT03149107	CHR-P (18-40); SIPS or SPI-A	1. Integrated Preventive Psychological Intervention plus N-Acetylcysteine; 2. Psychological stress management and N-Acetylcysteine; 3. Integrated Preventive Psychological Intervention and placebo; 4. Psychological stress management and placebo	Randomized (participant, investigator, outcomes assessor)	6 months	200	Transition to psychosis
Randomized Controlled Trial of Aspirin vs Placebo in the Treatment of Patients With the Clinical Risk Syndrome for Psychosis. NCT02047539	CHR-P (19-35); SIPS	1. Aspirin (2-Acetoxybenzoic acid); 2. placebo	Randomized (participant, care provider, investigator outcomes assessor)	12 weeks^(a)^	40	Symptoms improvement
Placebo-controlled Trial in Subjects at Ultra-high Risk for Psychosis With Omega-3 Fatty Acids in Europe (PURPOSE). NCT02597439	CHR-P (13-20); CAARMS	1. Omega-3 fatty acids; 2. placebo	Randomized (participant, care provider, investigator)	6 months	220	Transition to psychosis
Effects of Neurocognitive and Social Cognitive Remediation in Patients at Ultra-High Risk of Psychosis (FOCUS). NCT02098408	CHR-P (18-40); CAARMS	1. Standard treatment + cognitive remediation; 2. standard treatment	Randomized (investigator, outcomes assessor)	6 months	126	Cognitive functioning
Cognitive Behavioral Social Skills Training for Youth at Risk of Psychosis. NCT02234258	CHR-P (14-30); SIPS	1. Cognitive behavioral social skills; 2. psychoeducation	Randomized (outcomes assessor)	18 weeks	225	Social functioning
Decreasing Risk of Psychosis by Sulforaphane (DROPS Trial). NCT03932136	CHR-P (15-45); SIPS	1. Sulforaphane; 2. placebo	Randomized (participant, care provider, investigator, outcomes Assessor)	52 weeks	300	Transition to psychosis
Targeted Cognitive Training in Clinical High Risk (CHR) for Psychosis. NCT02404194	CHR-P (15-30); SIPS	1. Targeted cognitive training; 2. computer game	Radomized (participant, investigator, outcomes assessor)	10 weeks	76	Cognitive functioning
Minocycline and/or Omega-3 Fatty Acids Added to Treatment as Usual for At Risk Mental States (NAYAB). NCT02569307	CHR-P (16-35); CAARMS	1. Minocycline; 2. omega-3 fatty acids; 3. treatment as usual	Randomized (participant, care provider, investigator)	6 months	320	Transition to psychosis
The Staged Treatment in Early Psychosis Study (STEP). NCT02751632	CHR-P (15-25); CAARMS	staged treatment: 1. support and problem solving therapy; 2. cognitive behavioural case management; 3. cognitive behavioural case management plus fluoxetine; 4. cognitive behavioural case management plus placebo	Randomized (participant, investigator, outcomes assessor)	up to 12 months	340	Global functioning
Exercise and Markers of Medial Temporal Health in Youth at Ultra High-risk for Psychosis. NCT02155699	CHR-P (16-24); SIPS	1. Exercise 1; 2. exercise 2; 3. waiting list	Randomized (outcomes assessor)	3 months	45	Brain volume
Glutamate Reducing Interventions in Schizophrenia. NCT03321617	CHR-P (18-30); NA	1. pomaglumetad methionil 40 mg, 2. pomaglumetad methionil 80 mg, 3. pomaglumetad methionil 120 mg, 4. pomaglumetad methionil 160 mg	Randomized (participant, investigator, outcomes assessor)	2 weeks	50	Cerebral blood volume
Transcranial Direct Current Stimulation Coupled With Virtual Rehabilitation for Negative Symptoms in At-Risk Youth. NCT02951208	CHR-P (16-30); SIPS	1. Active Transcranial Direct Current Stimulation Coupled With Virtual Rehabilitation; 2. sham conditions	Randomized (participant, care provider, investigator outcomes assessor)	4 weeks	22	Symptoms improvement
Neurofeedback Processing Speed Training to Improve Social Functioning in Teenagers and Young Adults at Clinical High Risk for Psychosis. NCT03447548	CHR-P (12-25); SIPS	1. Neurofeedback processing speed training, 2. control	Radomized (participant, outcomes assessor)	NA	105	Cognitive functioning
A Phase II Randomised, Double-blind, Placebo-controlled Study to Evaluate the Efficacy, Safety, and Tolerability of Orally Administered BI 409306 During a 52-week Treatment Period as an Early Intervention in Patients With Attenuated Psychosis Syndrome. NCT03230097	DSM-5-APS (16-30); DSM-5	1. BI 409306; 2. placebo	Randomized (participant, investigator)	52 weeks	300	Time to remission

^(a)^not clear.

#### Oxytocin

Impairments of social cognition, which include emotional processing, theory of mind, and attributional style (82), are a primary cause of disability in psychosis (83) and respond poorly to current treatments. Emotional dysregulation and social cognition problems are also common in CHR-P individuals (84), are a key source of distress, and contribute to loss of functioning (85). Oxytocin, a neuropeptide, has numerous prosocial and antipsychotic-like effects in animals (86, 87). In healthy individuals, oxytocin promotes interpersonal trust (88), social interactions, and emotional bonding while decreasing arousal and aversion towards negative or threatening social stimuli (89) [for review see (90)]. In patients with psychosis, oxytocin has been shown to improve emotional recognition and social dysfunction and may ameliorate psychotic symptoms (91), although the hope of its potential as a treatment in established psychosis has been somewhat stifled by recent negative meta-analytic findings (92, 93). Nevertheless, neuroimaging studies demonstrate that oxytocin can modulate various indices of brain function, both task-specific and at rest, and in regions critically implicated in the onset of psychosis, such as limbic and midbrain brain regions (94–96). A randomized, double-blind, placebo-controlled, crossover acute-challenge MRI study has recently shown that oxytocin modulates hippocampal perfusion in CHR-P individuals (97), providing the first neurophysiological evidence for disease-target engagement. Further studies are ongoing that will better clarify the neurobiological effects of oxytocin in this patient population.

#### Cannabidiol

Cannabidiol is a major constituent of cannabis. In contrast to the psychoactive cannabinoid Δ9-tetrahydrocannabinol (THC), the non-psychoactive compound cannabidiol shows anxiolytic and potential antipsychotic properties. Early findings that administration of cannabidiol reduced psychotic symptoms in patients with established psychosis led to the suggestion that it may also have therapeutic potential in those at CHR-P (98, 99). Interest in cannabidiol is enhanced by its unique mechanism of action compared to established antipsychotics, as well as its distinct lack of serious adverse effects. A randomized controlled trial using cannabidiol and MRI in CHR-P individuals has recently been completed. In 33 CHR-P individuals and 19 healthy controls, cannabidiol was found to modulate activation of brain regions strongly implicated in the onset of psychosis during verbal learning, such as the striatum, medial temporal cortex, and midbrain (100). These early results support the view that cannabidiol may be an effective treatment strategy. Future large-scale randomized controlled trials involving cannabidiol are expected from different research centers worldwide.

### Simulation of the Living Meta-Analysis

**Figure 4:**
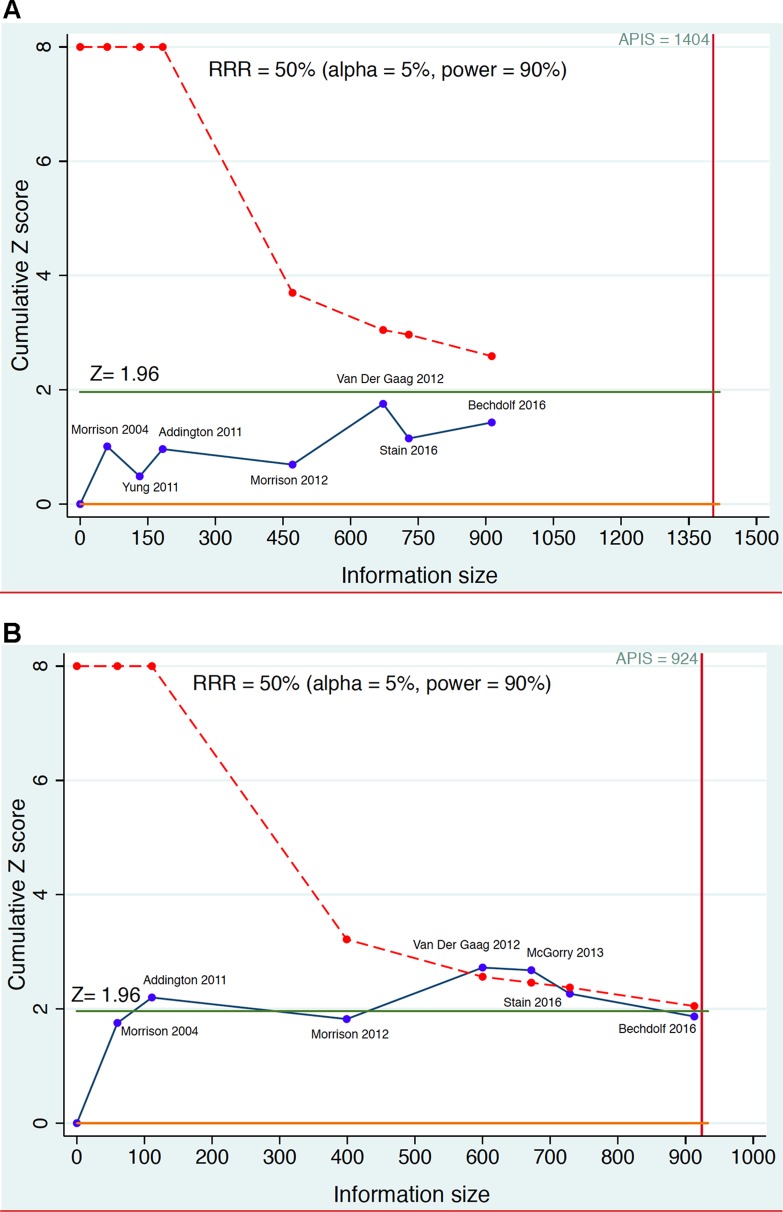
Example of sequential meta-analysis testing the preventive efficacy of CBT in CHR-P individuals. Plotted are the Randomized Controlled Trials of CBT vs Needs Based Intervention (NBI) in CHR-P patients that reported risk of psychosis onset at 6-month (part **A**) and 12 months (part **B**). The blue line represents the Z-value of each interim meta-analysis, the green line indicates the statistical significance threshold and the dotted red line the monitoring boundary. The red vertical line represents the *a priori* information size (APIS), i.e., the required sample size to detect a Relative Risk Reduction (RRR) = 0.5 with statistical power 0.9 on the risk of psychosis. Estimated using the package metacumbounds (101) and the previously published meta-analytical results (30).

**Figure 5:**
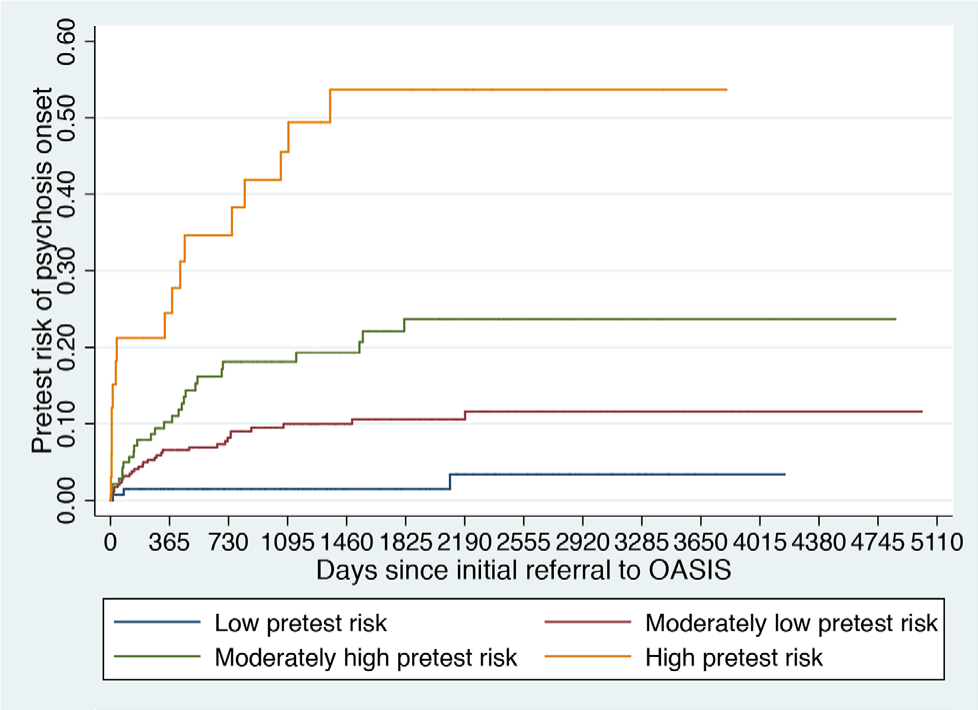
Clinical Stratification of Pretest Risk Enrichment in Individuals undergoing a Clinical High Risk assessment (102).

The results of the simulated living meta-analyses is provided in **Figures 4** and **5**.

**Figure 4** illustrates the theoretical results of a cumulative living meta-analysis investigating the efficacy of CBT versus needs-based intervention on the risk of developing psychosis at 6-months, for a treatment which can halve the risk of psychosis (Risk Ratio Reduction, RRR = 0.5). The a priori information size (APIS) plotted in **Figure 4** represents the sample size needed to investigate this effect, with an alpha of 0.05 and power of 0.9 (given the high uncertainty of the field the safest approach would be to plan a high power). The randomized controlled trials are analyzed in order of publication time, with the effect size of the (theoretical) living meta-analysis being updated every time a new trial is published. As shown in **Figure 4**, all updates of the living meta-analysis are reporting non-significant results that never cross the monitoring boundaries (lack of evidence), and that the APIS needed (given the minimal clinically relevant RRR, alpha and power) was not reached. Had a living meta-analysis been planned and performed, it would have shown that all published CBT trials were underpowered for testing the prevention of psychosis at 6-month follow-up under the above parameters (i.e. were false negatives). **Figure 4** investigates the preventive effect of CBT versus needs-based interventions in CHR-P individuals at 12 months. With the second trial there are significant results but a spurious effect because the curve does not cross the monitoring boundary (false positive result). The fourth and fifth trials produce significant results that cross the monitoring boundaries before the information sample is reached (true positive results). However, with the addition of the sixth and seventh trials the required APIS is almost met, the monitoring threshold is not crossed, and the result becomes non-significant (this could be a true negative result). Importantly, as noted above, these estimates relate to relatively large effect sizes (RR = 0.5). Current interventional CHR-P literature to date has not been powered to detect smaller effect sizes.

## Discussion

This is the first umbrella review of the evidence for preventive interventions in CHR-P individuals. Seven meta-analyses that were investigating preventive treatments in CHR-P individuals were included. None of them produced pooled effect sizes across psychological, pharmacological, or other types of interventions. The outcomes analyzed encompassed: risk of psychosis onset from a CHR-P state, acceptability of treatments, severity of attenuated positive and negative psychotic symptoms, depression, symptom-related distress, social functioning, general functioning, and quality of life. There was no evidence to favor any of the current preventive interventions over each—and any—other to improve any of these clinical outcomes. These meta-analyses were based on 20 randomized controlled trials. The vast majority of them (70%) defined the prevention of psychosis onset as their primary outcome (first-order issue) of interest. Several new trials are ongoing in this population, reflecting the high interest in this field. Simulation of a living meta-analysis did not change the interpretation of the current evidence.

The following sections interpret these findings in the context of the overall evidence for interventions in CHR-P individuals, the use of pooled vs treatment-specific effect sizes, the definition of primary and secondary outcomes in the published trials, and future recommendations to advance clinical research in this field.

### Evidence-Based Efficacy of Preventive Treatments for CHR-P Individuals

As noted in the introduction, it has been argued that the CHR-P paradigm has the capacity to i) identify people at incipient risk for psychosis, ii) improve their levels of distress and functioning, and iii) reduce their risk of progression to sustained psychotic disorder (32). It is true that the CHR-P paradigm has allowed the first prospective identification of individuals at risk of developing psychosis in clinical psychiatry. The achievements of the CHR-P paradigm have been extensively celebrated over the past years (3). At the same time, some important challenges have emerged. As illustrated by the trough of disillusionment phase in the Hype Cycle in **Figure 1**, the actual ability of specialized CHR-P clinics (4) to detect individuals at risk for psychosis is limited. An original study has estimated that only around 5% of individuals who will later develop a first episode of psychosis in secondary mental health care were detected by the local services at the time of their CHR-P stage (15, 103). Front-line youth mental health services, as opposed to specialized CHR-P clinics such as the OASIS, can be expected to detect more at-risk individuals. However, the limited evidence indicates that even youth mental health services can detect only 12% of first episode cases at the time of their CHR-P stage (104). It is therefore clear that we need to improve our ability to detect CHR-P individuals in secondary mental health care, primary care, and in the community. Some experimental approaches have been validated, which include automatic risk calculators for use in mental health trusts (57, 103, 105) or in the community (106).

Conversely, there is no evidence that current treatments are effective in improving the level of distress and functioning of CHR-P individuals. The most updated meta-analytical evidence summarized in **Table 2** shows no evidence to favor any preventive treatments over each (and any) other for improving any of the clinical outcomes that are relevant for CHR-P individuals: risk of developing a first episode of psychosis, acceptability of treatments, severity of attenuated positive or negative psychotic symptoms, depression, symptom-related distress, level of social functioning, level of general functioning, and quality of life. Although CHR-P patients may improve in their symptoms pre-post treatment, there are no substantial differences across interventions (which also includes control conditions). Importantly, although the meta-analyses presented in **Table 2** were conducted by different and independent research teams, their results converge. For example, two independent meta-analyses both concluded that there is no evidence to favor specific treatments over each other for improving attenuated positive psychotic symptoms in CHR-P individuals (58, 61). Unfortunately, there is no robust evidence that current treatments can improve functional levels in these patients. The most recent meta-analysis to have explored this outcome concluded that Cognitive Behavioral Therapy did not significantly improve social functioning at 6, 12, or 18 months; Omega-3 did not significantly improve social functioning at 6 or 12 months and Cognitive Remediation did not significantly improve social functioning at 2- to 3-month follow up (64). Lack of evidence for a symptomatic effect of preventive treatments in CHR-P individuals is also in line with our limited understanding of their mechanism of action at a neurobiological or psychological level (107). An independent pairwise meta-analysis published by the Cochrane group after completion of the current study has confirmed these findings. The study concluded that “there was no convincing, unbiased, high-quality evidence to suggest that any type of intervention is of value” for CHR-P people (108).

### Apples, Oranges, and the Production of Clinically Meaningless Pooled Effect-Sizes

A second issue, as detailed in the introduction (32), is the possibility that if the two recent network meta-analyses (30, 61) had pooled together psychosocial and pharmacological trials, statistically significant findings could have emerged (32). On a statistical and conceptual level, if the randomized controlled trials are all pooled together, there could be no “network” meta-analysis comparing specific nodes against each other but just a simple meta-analysis of the average efficacy of all treatments. On a pragmatic level, it is indeed true that these two meta-analyses (30, 61) did not even estimate a pooled effect size for all types of treatments, because this has little clinical interest and, as such, it was deliberately not planned in the *a priori* protocol of these studies (109). These meta-analyses were conducted to estimate the level of evidence with respect to specific clinical questions. Since preventive treatments for CHR-P individuals are highly heterogeneous, pooled effect sizes are clinically meaningless and cannot be used to inform treatment guidelines. Although a statistically significant pooled effect size may mean that interventions overall work, how would a clinician, who needs to decide on the best treatment to offer to a CHR-P patient, fare when interpreting a pooled effect size which has been estimated across pharmacological, psychological, and dietary interventions vs heterogeneous control conditions? Such an effect size would, at best, suggest that “any type of experimental treatment” is better/worse/similar than “any type of control condition”. This is some information (i.e. the intervention seems on average to work), but it is clearly useless from a clinical standpoint. Treatment guidelines need to be as specific as possible because clinicians, healthcare providers, and patients rely on them for clear direction and education on the best ways of adhering to them. Three previous aggregate pairwise meta-analyses were cited by the authors as support for pooling effect sizes across pharmacological and psychological categories of treatments (32). The first meta-analysis cited did not estimate any pooled effect sizes at all (26). Rather, this meta-analysis performed separate pairwise comparisons of specific preventive interventions, such as cognitive behavioral therapy vs supportive counselling (26). The second meta-analysis did compute significant pooled effect sizes and concluded that “receiving any of the focused treatment (53)” was associated with a reduced risk of psychosis onset. This clearly demonstrates the criticalities above: What exactly is a “focused treatment” and how should it be implemented in clinical routine? Because the authors could not answer such a question, they stated that their results did “not allow recommendation for any specific treatment” (53). This further demonstrates that pooled effect sizes in the CHR-P field are clinically meaningless and cannot inform treatment recommendations. It is, therefore, important to examine the efficacy of specific treatment effects in CHR-P individuals. Accordingly, the rationale for conducting the first randomized controlled trial in the CHR-P population was based on the need to examine more “specific interventions”—page 922 in Ref. (19). The third meta-analysis which was cited did compute a pooled effect size across cognitive behavioral therapy plus risperidone, olanzapine, omega-3, integrated psychotherapy (which included group skills training, cognitive remediation and multifamily psychoeducation in addition to cognitive behavioral therapy), and cognitive behavioral therapy compared to treatment as usual (52). In this case, the overall effect size was reported in **Figure 2** on page 60 (52) and it was inaccurately termed, in the figure itself, as “cognitive behavioral therapy” vs “treatment as usual”, ignoring the effect of risperidone, olanzapine, and dietary interventions (52). Furthermore, when this meta-analysis stratified the effect size by type of interventions, it concluded that “the subset of CBT-based interventions is associated with a pooled RR of 0.52 (95% CI = 0–79)”, and reported 95% confidence intervals which included the null hypothesis (RR = 1) (52). The lack of statistical significance could be due to the lower statistical power because of the stratification, yet this finding does not support a robust preventive effect of CBT-based interventions. This demonstrates that pooled effect sizes are not only of difficult clinical interpretability but also at very high risk of reporting biases.

Interventions for CHR-P are intrinsically highly heterogeneous because they include different therapeutic components. This is due to the fact that when interventions were originally introduced in the first randomized controlled trial, the strategy adopted was to “include the best-bet specific therapies in a single enhanced intervention package to determine whether it was possible to delay the onset of psychosis” (19). The subsequent randomized controlled trials have followed this approach by testing different packages, each of which was characterized by specific therapeutic components. This issue is evident, for example, in the case of different types of psychological therapies, which have been defined as “black boxes” (110). The additional and substantial problem with using pooled effect sizes in the CHR-P field is that the control group, traditionally termed as “treatment as usual” (52), is per se poorly standardized and largely dependent on local service configurations and the availability of specific resources or competences (67). For example, treatment as usual may encompass supportive psychotherapy primarily focusing on pertinent issues such as social relationships and vocational or family problems, case management, providing psychosocial assistance with accommodation, education or employment, brief family psychoeducation and support, medications other than antipsychotics, or clinical monitoring and crisis management (26). Network meta-analyses can be used to deconstruct the subcomponents of needs-based-interventions. Network meta-analyses offer additional benefits over standard pairwise analyses in that the comparative efficacy of specific interventions can be estimated and ranked, even when two treatments have never been compared directly head-to-head (111). Furthermore, network meta-analyses can improve the precision of estimates by allowing integration of both direct and indirect treatment effect estimates (112). The World Health Organization recommends network meta-analyses over pairwise meta-analyses as a basis for informing clinical guidelines (113). Therefore, network meta-analyses should be considered the highest level of evidence in CHR-P treatment guidelines (114). Because of these reasons, over recent years independent research teams have used network meta-analytical approaches to investigate the specific efficacy of preventive interventions for CHR-P individuals. Importantly, the current umbrella review demonstrates that none of them has produced pooled effect sizes (58, 60, 62, 64). This further provides independent validation that pooled effect sizes across different types of treatments should not be estimated when conducting treatment meta-analyses in the CHR-P field.

### The Opportunistic (In)Significance of Preventing the Onset of Psychosis

The third issue raised by some authors was that recent meta-analyses have focused on “second-order issues,” namely transition to psychosis in CHR-P individuals (32). The relegation of the prevention of psychosis to a second-order clinical outcome conflicts with the previous two decades of experimental research in this field. The extent to which randomized controlled trials in CHR-P individuals have prioritized prevention of psychosis, compared to other outcomes, is reported in **Table 2**. All twenty randomized controlled trials but four, corresponding to 70% of published trials, have focused on preventing the first onset of psychosis in CHR-P individuals as their primary outcome. Even the trials that did not primarily focus on preventing transition to psychosis still recommended investigation of whether the specific interventions could affect conversion rates (74) and modify the course of disease (72).

Until now, there has been a converging consensus in the CHR-P field that prevention of psychosis was the foremost outcome and the ultimate aim of the entire paradigm. Authors of randomized controlled trials in CHR-P individuals have declared that the goals of early detection are: 1) postponement or prevention of the transition to frank psychosis, 2) reduction of the duration of untreated psychosis to a minimum in patients who develop florid psychosis, and 3) prevention of delay to accessing mental health services (78). The authors also explicitly acknowledged that prevention of psychosis is the main goal (78). In line with this, outcomes other than the onset of psychosis have been poorly operationalized in the CHR-P field. For example, there is no clear definition for symptomatic remission or good outcomes in CHR-P patients. The importance of preventing the onset of psychosis in CHR-P individuals has been further endorsed worldwide and supported by consensus papers (3). Notably, the first randomized controlled trial in these patients aimed at determining whether it was possible to delay the onset of psychosis (19). The rationale given was overall pointing to the fact that this would be the most important way to alter the course of the disorder and eventually improve the lives of many patients. Such accumulating evidence clearly indicates that the prevention of psychosis has been “the” first-order issue in the CHR-P and not at all a second-order issue, as argued (32). It does not seem justified to downgrade the relevance of preventing psychosis in CHR-P individuals just because recent meta-analyses have not found robust evidence to favor specific preventive interventions (including also control conditions such as needs-based interventions). It has been argued that recent meta-analyses are “pessimistic” and have “distracted from the key message” of the research literature, which is “in need of positive findings pointing to better outcomes” (32). Evidence synthesis is conducted to test the robustness of findings on a determinate topic, and not to meet the needs of scientists and researchers. Reporting negative as well as positive findings is equally important. By systematically identifying and addressing gaps in knowledge, “negative” evidence syntheses such as these may, in fact, help to advance the field. For example, in taking a closer look, recent meta-analyses have concluded that owing to large confidence intervals, the actual efficacy of treatments for preventing psychosis is mostly indeterminate (30). Some effective signal in treatments’ efficacy may have been missed because of the large clinical heterogeneity (67) of the population being investigated. In this sense, these results are not at all pessimistic, but they rather call for a new generation of experimental research in this field (see below). Conversely, the claim that prevention of psychosis is a second-order issue could discourage future trials from exploring this relevant outcome and the current uncertainty in the meta-analytical estimates may remain unresolved. On a more conceptual level, there is some consensus that psychosis onset as defined categorically is an arbitrary concept (115–117) and that it should be better refined or complemented by other relevant outcomes, such as severity of attenuated symptoms, disability, and functional outcomes. Yet, prevention of psychosis should remain the cornerstone and the most important outcome for the CHR-P field, complemented by other outcomes. Downgrading—or at worst, dropping—such a primary outcome would be an indirect demonstration that current CHR-P research has approached the lowest depths of the through of disillusionment, with complete failure with respect to its ability to prevent psychosis. It would also prove that preventive psychiatry is different from other branches of medicine. For example, lack of effective treatments to prevent dementia (118) from a mild cognitive impairment stage has not triggered neurologists to claim that prevention of dementia itself should be downgraded to a second-order issue (instead it has become a priority for public health) (119).

### Towards the Slope of Enlightenment and the Next Generation of Experimental Therapeutics

As noted above, some authors have used recent findings to claim that the CHR-P paradigm should be completely abandoned. Such a claim seems too intransigent and not evidence-based. For example, by using the same criteria we should claim that integrated clinical services for first episode of psychosis should be dismantled, given that there is no evidence that they can reduce the duration of untreated psychosis (120) or prevent relapse (1). The absence of evidence is not evidence of absence (121). Furthermore, dismissing two decades of CHR-P research is not in line with the progressive nature of medical knowledge. As clarified in **Figure 1**, the trough of disillusionment often precedes the slope of enlightenment and the plateau of stable knowledge. The uncertain stage of knowledge which is typically associated with the trough of disillusionment is not specific to the CHR-P state; rather, it has also been observed in other branches of clinical medicine, such as cancer prevention (122). It is thus important to use our understanding of current limitations to advance knowledge, rather than hinder it.

#### The Essential Role of Risk Enrichment During Recruitment

The psychometric instruments traditionally used to ascertain the presence of a CHR-P state have been validated worldwide and, overall, have demonstrated excellent prognostic accuracy (AUC at 3 years: 0.9), comparable to that of other prognostic instruments employed in organic medicine (7). Yet, such an excellent prognostic accuracy is mostly due to the excellent sensitivity (96%) of CHR-P instruments to detect a state of risk for psychosis in help seeking patients who underwent some risk enrichment (i.e. pretest risk=15% at 3-year). Therefore, they are able to detect nearly all individuals who would develop psychosis, and consequently, nearly all individuals testing negative should be individuals who would not develop psychosis. In other words, a negative CHR-P assessment is associated with a very small probability of developing psychosis (1.56% at 3-year, negative likelihood ratio of 0.09) (7, 123). Conversely, the CHR-P instruments have a poor specificity (47%) and thus half of the individuals who would not develop psychosis might be false positives. Therefore, among individuals testing positive, most would not develop psychosis, or in other words, a positive CHR-P is still associated with a relatively small probability of developing psychosis (26% at 3-year, positive likelihood ratio of 1.82) (7, 123). Therefore, there is only a limited predictive gain in testing positive at a CHR-P assessment. As a consequence, CHR-P instruments should be used in samples that have already been enriched in their initial risk of psychosis and not as screening methods in the general population (see below) (7, 124). Otherwise, the global number of false positives would be so high, that most individuals with positive testing would be false positives (124). When individuals undergoing a CHR-P assessment are recruited from mental health services, they accumulate several risk factors for the disorder (6) which increase their level of risk to 15% at 3 years, compared to the 0.43% 3-year risk in the local age-matched general population (9, 102). This level of risk is also termed “pretest risk” because it is ascertained in the whole group of people undergoing a CHR-P assessment before the results of the assessment itself are known (123). The pretest risk in individuals recruited through mental health services (i.e. measured in naturalistic studies, excluding randomized controlled trials) is 15% at 3 years, worldwide (5). However, such an estimate is highly variable, with study estimates ranging from 3% to 49%, because it is based on unstandardized idiosyncratic recruitment strategies (5). When these individuals are assessed (tested), those meeting CHR-P criteria will have a 26% risk of developing psychosis at 3 years (1.7-fold increase from the pretest of 15%) and those not meeting CHR-P criteria will have a 1.56% risk of developing psychosis at 3 years (10-fold decrease from the pretest risk of 15%). As indicated in **Table 4**, assuming an alpha of 0.05, power of 90% (a higher power of 90% is recommended given the high uncertainty of this field; see **Table S1** for sample size estimations using 80% power), 2-sided test, it is possible to estimate the sample size which is required to test a new experimental treatment to prevent psychosis against treatment as usual (e.g. needs-based intervention). If the experimental treatment is able to halve the risk of psychosis (risk ratio = 0.5), 2,368 CHR-P individuals [conservatively using the 3% lower bound of the study estimates to avoid additional underpowered negative trials (31)] are required to complete the trial. After considering some attrition due to lost to follow-up (e.g. 20%), the final sample size would be of 2,842 CHR-P individuals. The problem is that this sample size is based on the pretest estimate from naturalistic studies that more likely represent the whole population seeking help at specialized CHR-P clinics. When CHR-P individuals detected by these specialized clinics are recruited into randomized controlled trials, it is likely that additional sampling biases would apply, further reducing the risk enrichment. For example, the push towards recruiting sufficient numbers of trial participants within a fixed period of time may lead to more intensive outreach campaign in the local community, which are well known to dilute the pretest risk (125). In other words, using unstructured recruitment strategies it may not be sufficient to recruit 2,842 CHR-P in new trials to be sufficiently powered to test preventive effects of magnitude 0.5. Furthermore, such a study would not be powered to test smaller effect sizes. If the efficacy of the preventive is lower, for example, if the experimental treatment is able to reduce the risk of psychosis onset by only 40% (risk ratio = 0.6) or 30% (risk ratio = 0.7), 3,942 and 7,436 CHR-P patients are needed to complete the trial (ignoring attrition), respectively. If individuals undergoing a CHR-P assessment are mostly recruited from the community, they will have accumulated less or no risk factors for psychosis (67) and their pretest risk would be 0.43% at 3 years. Following the estimates reported in **Table 4**, the sample size required for a similar randomized controlled trial would exceed 17,908 CHR-P individuals (ignoring attrition). In fact, CHR-P instruments do not work well when they are applied outside clinical samples that have already undergone some pretest risk enrichment (124, 125). As noted above, the way individuals are recruited (for undergoing a CHR-P assessment) drives the level of risk enrichment (5) and ultimately, impacts the statistical power of the trial. For example, the Neurapro trial has observed a risk of psychosis onset of about 14% at 3.4 years (29) in CHR-P individuals who had received the control condition, which suggests a pretest risk of 8.2% (we estimated the pretest risk of the sample as the transition risk of the control condition, assuming that there was no effect of needs based intervention). Under those circumstances, 796 CHR-P individuals would be needed to detect a 50% decrease in risk in the experimental condition, which exceeded the sample size employed by the trial. If there is some effect of the needs based intervention in the control condition, [as speculated by the authors (28)], then the pretest risk would be lower than that we estimated, and therefore the required sample size would be larger.

**Table 4:** Risk enrichment impacts statistical power and sample size for experimental therapeutic trials in CHR-P samples.

Sampling	Recruitment (pretest)^(e)^	Psychometric assessment (post-test)^(e)^		Total sample size excluding attrition ^(c)^		
Type of sample	Risk of psychosis at 3 years	Risk of psychosis at 3 years		Risks Ratio (Risk experimental treatment/Risk needs-based intervention		
		*CHR+* *^(a)^*	*CHR-* *^(b)^*	0.5	0.6	0.7
Pretest risk in people undergoing CHR assessment outside randomized clinical trials	0.03^(d)^-0.49	0.051	0.003	2368	3942	7436
General population	0.004	0.007	< 0.001	17908	29844	56360
Neurapro trial -control arm	0.082	0.140	0.008	796	1324	2488
Pretest risk stratification						
*low pretest risk*	0.014	0.026	0.001	4730	7880	14874
*moderately low pretest risk*	0.100	0.168	0.010	648	1076	2020
*moderately high pretest risk*	0.181	0.287	0.020	336	554	1036
*high pretest risk*	0.456	0.604	0.070	104	170	310

^(a)^LR+ = 1.82. ^(b)^LR- = 0.09. ^(c)^alpha = 0.05; power 90%; 2-sided; allocation ratio = 1. The sample sizes reported in the table indicate the individuals who should complete the trial (to estimate the baseline sample, attrition should be considered). ^(d)^the average is 0.15 but because it depends on unstandardized idiosyncratic recruitment strategies, it is highly variable ranging from 3% to 49% depending on the study (5). It tends to be on the lower side when the recruitment focuses on children and adolescent populations, intermediate when the recruitment focuses on primary care settings and higher when the recruitment focuses on secondary care. In this table to allow a conservative estimate of the sample size required we use only the lower bound of 0.03. ^(e)^Post-test probability = LR*pretest probability/[(1-pretest probability)+(pretest probability*LR)].

It is therefore possible that the low level of risk for psychosis decreased the statistical power of the trial to detect small signal effects associated with the experimental treatments. Lack of statistical power because of a poor level of psychosis risk may actually be one of the causes of the negative randomized controlled trials in this population (31) and of the associated large 95% confidence intervals that have been observed in the last network meta-analysis (30). The main problem is that recruitment strategies in this field are idiosyncratic and poorly standardized and as such, it is not possible to control the level of pretest risk enrichment. This is particularly concerning in the case of recruitment into trials, which, as noted above, introduces additional selection biases and may further dilute the risk enrichment. For example, likely because of more intense outreach campaigns in the community, the actual risk of psychosis in CHR-P samples has been declining from 29% (2012 (126)) to 20% (2016 (12)) worldwide. Interestingly, there are exceptions to this phenomenon, such as the Outreach and Support in South London (OASIS) CHR-P service (4), where transition risk has not declined over time. This is again due to the fact that recruitment strategies have, overall, maintained a stable pretest risk enrichment (127). Ultimately, innovative strategies are needed to ensure that a sufficient level of risk enrichment is obtained to allow adequate statistical power in CHR-P recruited into future trials. A possible solution could be to apply pretest risk stratification algorithms that have been developed and validated for this population using machine learning methods (**Figure 5**) (102).

For example, pretest risk estimation algorithms that are based on the source of referral to CHR-P clinics and ethnicity can be used to stratify individuals into four classes of risk enrichment: low risk (1% risk at 3 years, ≈21% of the CHR-P population), moderately low risk (10% at 3 years, ≈53% of the CHR-P population), moderately high (18% at 3 years, ≈21% of the CHR-P population), and high (46% at 3 years, ≈5% of the CHR-P population, **Figure 5**). If this simple tool is applied to individuals recruited for a CHR-P assessment it may be possible to control risk enrichment and avoid its high variability. Furthermore, stratification may inform the subsequent phases. For example, those who are at low risk are to be screened out from trial eligibility, a pretest risk enrichment of at least 10%—or more—would be ensured across all participating sites (**Figure 5**). Under those circumstances, a total sample of 648 CHR-P individuals (ignoring attrition) would guarantee sufficient statistical power (90%) to test treatment effects that can halve the risk of developing psychosis. Alternatively, those with a moderately low pretest risk can be subjected to further testing to refine the prognostic accuracy (128). Controlling pretest risk enrichment through the recruitment phase is expected to mitigate the existing challenges and facilitate the slope of enlightenment phase (**Figure 1**).

#### Stratification and Precision Medicine

As noted above, the lack of evidence to favor specific preventive treatments over any others should be the basis to promote further research in this field, rather than to abandon it. For example, it is likely that a one-size-fits-all treatment approach in CHR-P populations is not effective and that some treatments may work for specific subgroups of patients. There are several lines of evidence to support this. First, some meta-analyses included in the current umbrella review report large 95% confidence intervals, which indicate high heterogeneity across treatments. Some of them may actually be effective. For example, the network meta-analysis exploring the effect of treatments on the prevention of psychosis has suggested that the integrated psychological intervention developed by Bechdolf et al. (22) has the largest effect size for reducing the risk of psychosis onset compared to needs-based interventions (Odds Ratio = 0.04) (30). Although the 95% confidence intervals included the null hypothesis, this treatment could be the focus of the next generation of therapeutic trials. An important note is that current meta-analytical evidence did not favor any treatment compared to each other (including control conditions such as needs-based-intervention). As such, it is possible that needs-based-intervention per se was associated with improved outcomes, diluting the potential comparative efficacy of other interventions. Another example comes from the failure of some randomized controlled trials, such as the NEURAPRO (omega-3) when tested in an unstratified CHR-P sample (28). This has naturally led to the suggestion that omega-3 might be more efficacious in those individuals who specifically have low levels of membrane fatty acids at baseline (129). This makes sense from a pathophysiological perspective—psychosis is a heterogeneous disorder with likely many different neurobiological ‘paths’ and risk factors (67), which may perturb an individual’s neural circuitry in various (innumerable) ways and which ultimately presents as psychosis (107).

**Table 5** provides a clinical interpretation of the evidence for selecting preventive interventions for CHR-P individuals.

**Table 5:** Clinical interpretation of the current evidence for selecting a preventive intervention for CHR-P individuals.

Currently, no reliable recommendation can be made regarding whether specific interventions (e.g. psychological interventions, medications, dietary interventions, needs-based interventions) are more effective compared to each other for the prevention of psychosis in CHR-P individuals.
Consequently, the safest approach is recommended, that is needs-based interventions and psychological interventions over antipsychotics, because the latter are not more efficacious than other options and have known side effects.
The selection of these two interventions should be based on factors such as the characteristics of each individual. These can include patients’ preferences (e.g., some patients may prefer psychological interventions over needs-based interventions), social circumstances (e.g. needs based interventions which include housing/vocational support may be suited for patients for whom these issues represent the presenting complaint), nature of symptoms (e.g. psychological interventions may be indicated for those presenting with cognitive biases in addition to attenuated psychotic symptoms), predicted risk (e.g. those presenting with brief and limited intermittent psychotic symptoms may need psychological treatments beyond needs-based interventions) or the local availability of each intervention.
Some suggestions can be made on the basis of differences that, even if non-statistically significant, had at least a moderate effect size (Odds Ratio > 2.5 for preventing the onset of psychosis, or Cohen’s d > 0.5 for reducing symptoms).
• The most efficacious intervention for preventing psychosis onset *could* be Integrated Psychological Interventions (IPI), and the second most efficacious *could* be Cognitive Behavioral Therapy (van der Gaag protocol) combined with needs-based interventions.
• Omega-3 and CBT *could* be more efficacious than IPI for improving attenuated positive psychotic symptoms.
• N-methyl-d-aspartate receptor modulators *could* be more efficacious than needs-based interventions alone, CBT and family therapy for improving attenuated negative symptoms.
• Importantly, none of these differences reached statistical significance.
Finally, it will be essential to consult the results of new and forthcoming studies as they emerge. In this regard, living meta-analyses could provide new evidence earlier than updating a conventional meta-analysis.

Second, there is converging evidence indicating that the CHR-P group is clinically heterogeneous. For example, there is high heterogeneity in the level of risk for psychosis across the three CHR-P subgroups of the attenuated psychotic symptoms, brief and limited intermittent psychotic symptoms, and genetic risk and deterioration syndrome (12, 116, 117). In particular, those meeting the brief and limited intermittent psychotic symptoms criteria have a very high risk of developing persistent psychotic disorders. Recent studies also demonstrate that these patients are those in highest clinical need but receive inadequate care (130, 131). Such a heterogeneity calls for a revision of the CHR-P paradigm which should include clinical stratification across these three subgroups (10, 17). Ongoing international consortia such as PSYSCAN, PRONIA, and NAPLS have already started delivering precision medicine tools for stratifying CHR-P individuals and may permit an individualized prediction of their outcomes (57, 132, 133). These consortia are adopting a multimodal prediction approach which is spanning clinical, neurocognitive, neurobiological, as well as genetic predictors (134) to improve the individualized prognosis of outcomes in this population.

#### Individual Participant Data Living Meta-Analyses

Another precision medicine approach that is expected to advance knowledge is to conduct further evidence synthesis studies such as individual-participant data level network meta-analyses. The use of individual data as opposed to aggregate-level meta-analysis would also allow analyzing the effect of different individual factors (including the CHR-P subgroups) and development of evidence-based prognostic algorithms to forecast the likelihood of treatment response at the individual subject level based on these factors. It is expected that these individual-participant data meta-analyses will identify specific subgroups of CHR-P individuals for whom current treatments (i.e. the integrated psychological intervention) may already be effective. A relevant issue (highlighted in **Table 5**) is that the future publication of a single study with robust evidence of efficacy may significantly change the level of evidence for preventive interventions in psychosis. As indicated in **Table 3** and in the section below, novel compounds for this patient population are under investigation, and it is thus expected that new results will be released over the next few years. This would be the ideal context to conduct living meta-analyses. To illustrate their methodological approach and the potential impact in the field we have simulated a living meta-analysis. The simulation showed no robust evidence to favor current CBT treatments for the prevention of psychosis. Prospectively planned living systematic reviews and meta-analyses are thus expected in this field. The feasibility and sustainability of living systematic reviews can be supported by the synergic interaction of human effort and machine automation (135). A recent empirical study has demonstrated that prospectively planned living network meta-analyses produced strong evidence against the null hypothesis more often—and earlier—than conventional pairwise meta-analyses (39).

#### Innovative Youth Mental Health Services and Advanced Trial Designs

Additional important factors that may support the slope of enlightenment are the development of youth mental health services and innovative trial designs. The high prevalence of mental health problems among young people, their negative impact on outcomes, as well as their significant financial and societal cost, emphasizes the need to improve broad mental health care in children and young people (136). The “No Health Without Mental Health” report by the UK Government recognized that only a life-course approach would allow fulfilment of such an objective, and highlighted the importance of the early years (137). Similarly, the “Future in Mind” report by NHS England highlights the pressing need (by 2020) for a holistic approach, including better access and support for front-line staff, and the adoption of innovative integrated youth mental health approaches which depart from the current tier system which is split between Child and Adolescent Mental Health Services and Adult Mental Health Services (138). The OASIS model presented above already provides integrated care across child and adolescent and adult mental health services for CHR-P individuals aged 14–35. Notably, the CHR-P services platform has been recently implemented nationwide in some countries, demonstrating scalable impact for taking care of children and young adults. Therefore, it may be possible to leverage these CHR-P templates in order to refine the next generation of youth-friendly mental health services which target the needs of adolescents and young adults experiencing early stages of other mental disorders, such as depression (139) and bipolar disorders (140, 141). Some integrated models of care have already capitalized on the CHR-P platform to broaden their horizons and target the wider mental health of children and young adults. In 2006, following a campaign that was led by leaders in mental health, the early intervention model for psychosis was then expanded to other diagnoses (e.g., mood, personality, eating, and substance use disorders) through the creation of Headspace in Australia (142). Headspace is a government-funded initiative that provides youth-friendly, stigma-free early intervention services in a ‘one-stop shop’ location to 12–25-year olds with emerging mental health and substance use disorders (142). Within this context, mental healthcare is multidisciplinary, integrated, delivered in a single setting and is centered on the needs of young people along with their families (143). The youth mental health reform achieved in Australia has permeated to other regions of the world, with the UK, Ireland, Canada, USA, Europe, and Asia adopting similar, culturally appropriate models (143, 144).

Youth mental health services may offer some additional clinical research advantages because they fully embed the clinical staging model for the emergence of several mental disorders (1, 145). In turn, this would facilitate stepped clinical care. For example, by adopting subsequent prognostic assessments (8) in young people at risk for emerging mental disorders, it may be possible to refine the prediction of their longitudinal outcomes while optimizing the logistic resources and translational impact (128). The stepped prognosis can be associated with stepped care, which targets the level of need and risk presented by each person. Adaptive clinical trials represent an innovative and flexible method, allowing pre-specified modifications to the design or statistical procedures of an on-going trial depending on the data generated from it. Such adaptive design trials can boost clinical research by cutting costs and time, and more importantly, by adapting to the clinical stages of the stepped care model implemented in youth mental health services. The first adaptive design clinical trial, the Staged Treatment in Early Psychosis Study (STEP) (146), is ongoing and more are expected to follow.

#### New Experimental Therapeutics

As indicated in our latest network meta-analyses (30, 61) and in **Table 3**, innovative experimental therapeutics in CHR-P individuals are under development. These compounds, if used along with the above strategies, are expected to advance knowledge in this field.

In summary, the slope of enlightenment and future advancements of knowledge in this field could be facilitated by controlling pretest risk enrichment in samples undergoing CHR-P assessment, stratifying CHR-P samples to allow precision medicine approaches, conducting Individual Participant Data and living meta-analyses, adopting innovative youth mental health services and advanced trial designs, and testing novel experimental therapeutics.

## Conclusions

This umbrella review found no convincing evidence for superior efficacy of any current preventive treatment, compared to each—and every—other, on the risk of psychosis onset, acceptability of treatments, severity of attenuated positive and negative psychotic symptoms, depression, symptom-related distress, social functioning, general functioning, or quality of life in CHR-P individuals. Current interventional literature in CHR-P population has been only powered to detect large effect sizes for preventive treatments. Prevention of psychosis from a CHR-P state has been and should remain the (first order) primary outcome of interventional research, refined and complemented by other clinically meaningful outcomes. The stagnation of knowledge in the field should promote innovative and collaborative research efforts, rather than give us cause to abandon it. Advancements will most likely be associated with the ability to deconstruct the high heterogeneity of CHR-P populations. This would require the estimation of treatment-specific effect sizes through individual participant data meta-analyses, controlling risk enrichment during recruitment, and embedding stratification and precision medicine methods within new youth mental health services that can accommodate staged care, stepped prognosis, and advanced trial designs. Several experimental therapeutics studies in CHR-P individuals are also ongoing. The strategies which could facilitate reaching the plateau of knowledge in CHR-P field are summarized in Gartner Hype Cycle (**Figure 1**). We hope that these innovations will ultimately pave the way toward a future plateau of knowledge and refined preventive treatments that could result in tangible benefits for clinicians, researchers, and, more importantly, for patients and their families.

## Data Availability Statement

All datasets analysed for this study are included in the article.

## Author Contributions

PF-P designed the study, CD and JR conducted the analyses, MS, NB, AM, MK-A, JS drafted the manuscript along with the other authors. All authors contributed to the interpretation of the results. The authors have approved the current version of the manuscript.

## Funding

This study was supported by the King’s College London Confidence in Concept award from the Medical Research Council (MRC) (MC_PC_16048) to PF-P. These funding bodies had no role in the design of the study, collection, and analyses.

## Conflict of Interest

The authors declare that the research was conducted in the absence of any commercial or financial relationships that could be construed as a potential conflict of interest.

## References

[1] Fusar-Poli P McGorry PD Kane JM Improving outcomes of first-episode psychosis: an overview. World Psychiatry (2017) 16:251–65. 10.1002/wps.20446 PMC5608829 28941089

[2] Oliver D Radua J Reichenberg A Uher R Fusar-Poli P Psychosis Polyrisk Score (PPS) for the detectio of individuals at risk and the prediction of their outcomes. Front Psychiatry (2019) 10:174. 10.3389/fpsyt.2019.00174 31057431 PMC6478670

[3] Fusar-Poli P Borgwardt S Bechdolf A Addington J Riecher-Rossler A Schultze-Lutter F The psychosis high-risk state: a comprehensive state-of-the-art review. JAMA Psychiatry (2013) 70:107–20. 10.1001/jamapsychiatry.2013.269 PMC4356506 23165428

[4] Fusar-Poli P Byrne M Badger S Valmaggia LR McGuire PK Outreach and support in south London (OASIS), 2001-2011: ten years of early diagnosis and treatment for young individuals at high clinical risk for psychosis. Eur Psychiatry (2013) 28:315–26. 10.1016/j.eurpsy.2012.08.002 23137782

[5] Fusar-Poli P Schultze-Lutter F Cappucciati M Rutigliano G Bonoldi I Stahl D The dark side of the moon: meta-analytical impact of recruitment strategies on risk enrichment in the clinical high risk state for psychosis. Schizophr Bull (2016) 42:732–43. 10.1093/schbul/sbv162 PMC4838090 26591006

[6] Fusar-Poli P Tantardini M De Simone S Ramella-Cravaro V Oliver D Kingdon J Deconstructing vulnerability for psychosis: meta-analysis of environmental risk factors for psychosis in subjects at ultra high-risk. Eur Psychiatry (2017) 40:65–75. 10.1016/j.eurpsy.2016.09.003 27992836

[7] Fusar-Poli P Cappucciati M Rutigliano G Schultze-Lutter F Bonoldi I Borgwardt S At risk or not at risk? A meta-analysis of the prognostic accuracy of psychometric interviews for psychosis prediction. World Psychiatry (2015) 14:322–32. 10.1002/wps.20250 PMC4592655 26407788

[8] Fusar-Poli P Hijazi Z Stahl D Steyerberg EW The science of prognosis in psychiatry: a review. JAMA Psychiatry (2018) 75:1289–97. 10.1001/jamapsychiatry.2018.2530 30347013

[9] Fusar-Poli P Davies C Bonoldi I A case of a college student presenting With mild mental health problems. JAMA Psychiatry (2018) 75:1298–9. 10.1001/jamapsychiatry.2018.2486 30347005

[10] Fusar-Poli P The Clinical High-Risk State for Psychosis (CHR-P), Version II. Schizophr Bull (2017) 43(1):44–7. 10.1093/schbul/sbw158 PMC5216870 28053129

[11] Fusar-Poli P Rocchetti M Sardella A Avila A Brandizzi M Caverzasi E Disorder, not just state of risk: meta-analysis of functioning and quality of life in people at high risk of psychosis. Br J Psychiatry (2015) 207:198–206. 10.1192/bjp.bp.114.157115 26329563

[12] Fusar-Poli P Cappucciati M Borgwardt S Woods SW Addington J Nelson B Heterogeneity of psychosis risk within individuals at clinical high risk: a meta-analytical stratification. Jama Psychiatry (2016) 73:113–20. 10.1001/jamapsychiatry.2015.2324 26719911

[13] Fusar-Poli P Rutigliano G Stahl D Davies C De Micheli A Ramella-Cravaro V Long-term validity of the At Risk Mental State (ARMS) for predicting psychotic and non-psychotic mental disorders. Eur Psychiatry (2017) 42:49–54. 10.1016/j.eurpsy.2016.11.010 28212505

[14] Webb JR Addington J Perkins DO Bearden CE Cadenhead KS Cannon TD Specificity of incident diagnostic outcomes in patients at clinical high risk for psychosis. Schizophr Bull (2015) 41:1066–75. 10.1093/schbul/sbv091 PMC4535651 26272875

[15] Fusar-Poli P Extending the benefits of indicated prevention to improve outcomes of first-episode psychosis. JAMA Psychiatry (2017) 74:667–8. 10.1001/jamapsychiatry.2017.1009 28538947

[16] Gartner I Gartner Hype Cycle. (2018).

[17] Fusar-Poli P The Hype Cycle of the clinical high risk state for psychosis: the need of a refined approach. Schizophr Bull (2018). 10.1093/schbul/sbw158 PMC5216870 28053129

[18] Chen JH Asch SM Machine Learning and prediction in medicine - beyond the peak of inflated expectations. N Engl J Med (2017) 376:2507–9. 10.1056/NEJMp1702071 PMC5953825 28657867

[19] McGorry PD Yung AR Phillips LJ Yuen HP Francey S Cosgrave EM Randomized controlled trial of interventions designed to reduce the risk of progression to first-episode psychosis in a clinical sample with subthreshold symptoms. Arch Gen Psychiatry (2002) 59:921. 10.1001/archpsyc.59.10.921 12365879

[20] McGlashan TH Zipursky RB Perkins D Addington J Miller T Woods SW Randomized, double-blind trial of olanzapine versus placebo in patients prodromally symptomatic for psychosis. Am J Psychiatry (2006) 163:790–9. 10.1176/ajp.2006.163.5.790 16648318

[21] Morrison AP French P Walford L Lewis SW Kilcommons A Green J Cognitive therapy for the prevention of psychosis in people at ultra-high risk: randomised controlled trial. Br J Psyc (2004) 185:291–7. 10.1192/bjp.185.4.291 15458988

[22] Bechdolf A Wagner M Ruhrmann S Harrigan S Putzfeld V Pukrop R Preventing progression to first-episode psychosis in early initial prodromal states. Br J Psychiatry (2012) 200:22–9. 10.1192/bjp.bp.109.066357 22075649

[23] Amminger GP Schäfer MR Papageorgiou K Klier MC Cotton MS Harrigan MS Long-Chain omega-3 Fatty Acids for Indicated Prevention of Psychotic Disorders. Arch Gen Psychiatry (2010) 67:146–54. 10.1001/archgenpsychiatry.2009.192 20124114

[24] NICE Psychosis and schizophrenia in adults: prevention and management. National Institute for Clinical Excellence (2014). 32207892

[25] Schmidt SJ Schultze-Lutter F Schimmelmann BG Maric NP Salokangas RKR Riecher-Rössler A EPA guidance on the early intervention in clinical high risk states of psychoses. Eur Psychiatry (2015) 30:388–404. 10.1016/j.eurpsy.2015.01.013 25749390

[26] Stafford MR Jackson H Mayo-Wilson E Morrison AP Kendall T Early interventions to prevent psychosis: systematic review and meta-analysis. BMJ (2013) 346:f185. 10.1136/bmj.f185 23335473 PMC3548617

[27] Morrison AP French P Stewart SL Birchwood M Fowler D Gumley AI Early detection and intervention evaluation for people at risk of psychosis: multisite randomised controlled trial. BMJ (2012) 344:e2233. 10.1136/bmj.e2233 22491790 PMC3320714

[28] McGorry PD Nelson B Markulev C Yuen HP Schafer MR Mossaheb N Effect of omega-3 polyunsaturated fatty acids in young people at ultrahigh risk for psychotic disorders: the NEURAPRO randomized clinical trial. JAMA Psychiatry (2017) 74:19–27. 10.1001/jamapsychiatry.2016.2902 27893018

[29] Nelson B Amminger GP Yuen HP Markulev C Lavoie S Schafer MR NEURAPRO: a multi-centre RCT of omega-3 polyunsaturated fatty acids versus placebo in young people at ultra-high risk of psychotic disorders-medium-term follow-up and clinical course. NPJ schizophrenia (2018) 4:11. 10.1038/s41537-018-0052-x 29941938 PMC6018097

[30] Davies C Cipriani A Ioannidis JPA Radua J Stahl D Provenzani U Lack of evidence to favor specific preventive interventions in psychosis: a network meta-analysis. World Psychiatry (2018) 17:196–209. 10.1002/wps.20526 29856551 PMC5980552

[31] Fusar-Poli P Negative Psychosis Prevention Trials. JAMA Psychiatry (2017) 74(6):651. 10.1001/jamapsychiatry.2017.0185 28403388

[32] Nelson B Amminger GP McGorry PD Recent meta-analyses in the clinical high risk for psychosis population: clinical interpretation of findings and suggestions for future research. Front Psychiatry (2018) 9:502. 10.3389/fpsyt.2018.00502 30369889 PMC6194228

[33] Fusar-Poli P Radua J Ten simple rules for conducting Umbrella Reviews. Evid Based Ment Health (2018) 21(3):95–100. 10.1136/ebmental-2018-300014 30006442 PMC10270421

[34] Riley RD Lambert PC Abo-Zaid G Meta-analysis of individual participant data: rationale, conduct and reporting. BMJ (2010) 340:c221. 10.1136/bmj.c221 20139215

[35] Shea BJ Hamel C Wells GA Bouter LM Kristjansson E Grimshaw J AMSTAR is a reliable and valid measurement tool to assess the methodological quality of systematic reviews. J Clin Epidemiol (2009) 62:1013–20. 10.1016/j.jclinepi.2008.10.009 19230606

[36] Cipriani A Zhou X Del Giovane C Hetrick SE Qin B Whittington C Comparative efficacy and tolerability of antidepressants for major depressive disorder in children and adolescents: a network meta-analysis. Lancet (2016) 388:881–90. 10.1016/S0140-6736(16)30385-3 27289172

[37] Jadad AR Cook DJ Jones A Klassen TP Tugwell P Moher M Methodology and reports of systematic reviews and meta-analyses: a comparison of Cochrane reviews with articles published in paper-based journals. JAMA (1998) 280:278–80. 10.1001/jama.280.3.278 9676681

[38] Shojania KG Sampson M Ansari MT Ji J Doucette S Moher D How quickly do systematic reviews go out of date? A survival analysis. Ann Internal Med (2007) 147:224–33. 10.7326/0003-4819-147-4-200708210-00179 17638714

[39] Nikolakopoulou A Mavridis D Furukawa TA Cipriani A Tricco AC Straus SE Living network meta-analysis compared with pairwise meta-analysis in comparative effectiveness research: empirical study. BMJ (2018) 360:k585. 10.1136/bmj.k585 29490922 PMC5829520

[40] Bragge P Clavisi O Turner T Tavender E Collie A Gruen RL The Global Evidence Mapping Initiative: scoping research in broad topic areas. BMC Med Res Methodol (2011) 11:92. 10.1186/1471-88-11-92 21682870 PMC3141802

[41] Elliott JH Turner T Clavisi O Thomas J Higgins JP Mavergames C Living systematic reviews: an emerging opportunity to narrow the evidence-practice gap. PLoS Med (2014) 11:e1001603. 10.1371/journal.pmed.1001603 24558353 PMC3928029

[42] Elliott JH Synnot A Turner T Simmonds M Akl EA McDonald S Living systematic review: 1. Introduction-the why what, when, and how. J Clin Epidemiol (2017) 91:23–30. 10.1016/j.jclinepi.2017.08.010 28912002

[43] Lan K Demets D Discrete sequential boundaries for clinical trials. Biometrika (1983) 70:659e63. 10.2307/2336502

[44] Pogue JM Yusuf S Cumulating evidence from randomized trials: utilizing sequential monitoring boundaries for cumulative meta-analysis. Control Clin Trials (1997) 18:580–93discussion 661–6. 10.1016/S0197-2456(97)00051-2 9408720

[45] Brok J Thorlund K Wetterslev J Gluud C Apparently conclusive meta-analyses may be inconclusive–Trial sequential analysis adjustment of random error risk due to repetitive testing of accumulating data in apparently conclusive neonatal meta-analyses. Int J Epidemiol (2009) 38:287–98. 10.1093/ije/dyn188 18824466

[46] Wetterslev J Thorlund K Brok J Gluud C Trial sequential analysis may establish when firm evidence is reached in cumulative meta-analysis. J Clin Epidemiol (2008) 61:64–75. 10.1016/j.jclinepi.2007.03.013 18083463

[47] Nikolakopoulou A Mavridis D Egger M Salanti G Continuously updated network meta-analysis and statistical monitoring for timely decision-making. Stat Methods Med Res (2018) 27:1312–30. 10.1177/0962280216659896 PMC5863798 27587588

[48] Cook JA Hislop J Altman DG Fayers P Briggs AH Ramsay CR Specifying the target difference in the primary outcome for a randomised controlled trial: guidance for researchers. Trials (2015) 16:12. 10.1186/s13063-014-0526-8 25928502 PMC4302137

[49] Simmonds M Salanti G McKenzie J Elliott JN Living Systematic Review. Living systematic reviews: 3. Statistical methods for updating meta-analyses. J Clin Epidemiol (2017) 91:38–46. 10.1016/j.jclinepi.2017.08.008 28912004

[50] Higgins JP Whitehead A Simmonds M Sequential methods for random-effects meta-analysis. Stat Med (2011) 30:903–21. 10.1002/sim.4088 PMC3107948 21472757

[51] Michel C Toffel E Schmidt SJ Eliez S Armando M Solida-Tozzi A [Detection and early treatment of subjects at high risk of clinical psychosis: Definitions and recommendations]. Encephale (2017) 43:292–7. 10.1016/j.encep.2017.01.005 28347521

[52] van der Gaag M Smit F Bechdolf A French P Linszen DH Yung AR Preventing a first episode of psychosis: meta-analysis of randomized controlled prevention trials of 12 month and longer-term follow-ups. Schizophr Res (2013) 149:56–62. 10.1016/j.schres.2013.07.004 23870806

[53] Preti A Cella M Randomized-controlled trials in people at ultra high risk of psychosis: a review of treatment effectiveness. Schizophr Res (2010) 123:30–6. 10.1016/j.schres.2010.07.026 20727717

[54] Kelly C Hadjinicolaou AV Holt C Agius M Zaman R Meta-analysis of medical and non-medical treatments of the prodromal phase of psychotic illness in at-risk mental states. Psychiatr Danub (2010) 22 (Supp 1):56–62. 21057405

[55] Deas G Kelly C Hadjinicolaou AV Holt C Agius M Zaman R An update on: meta-analysis of medical and non-medicaltreatments of the prodromal phase of psychotic illness in at risk mental states. Psychiatr Danub (2016) 28 (Supp 1):31–8. 27663802

[56] Marshall M Rathbone J Early intervention for psychosis. In: Marshall M, editor. Cochrane Database of Systematic Reviews. John Wiley & Sons Ltd, (2011). 10.1002/14651858.CD004718.pub3

[57] Fusar-Poli P Werbeloff N Rutigliano G Oliver D Davies C Stahl D Transdiagnostic risk calculator for the automatic detection of individuals at risk and the prediction of psychosis: second replication in an independent national health service trust. Schizophr Bull (2019) 45(3):562–570. 10.1093/schbul/sby070 29897527 PMC6483570

[58] Devoe DJ Farris MS Townes P Addington J Attenuated psychotic symptom interventions in youth at risk of psychosis: a systematic review and meta-analysis. Early Interv Psychiatry (2019) 13(1):3–17. 10.1111/eip.12677 29749710 PMC6230498

[59] Stafford MR Jackson H Mayo-Wilson E Morrison AP Kendall T Errata: Early interventions to prevent psychosis: systematic review and meta-analysis. BMJ (2013) 346:f762–2. 10.1136/bmj.f762 PMC3548617 23335473

[60] Devoe J Peterson A Addington J Devoe DJ Peterson A Addington J Negative symptom interventions in youth at risk of psychosis: a systematic review and network meta-analysis. Schizophr Bull (2018) 44:463. 10.1093/schbul/sbx193 Erratum to. 29069511 PMC6007754

[61] Davies C Radua J Cipriani A Stahl D Provenzani U McGuire P Efficacy and acceptability of interventions for attenuated positive Psychotic Symptoms in Individuals at Clinical High Risk of Psychosis: A Network Meta-Analysis. Front Psychiatry (2018) 9:187. 10.3389/fpsyt.2018.00187 29946270 PMC6005890

[62] Devoe DJ Peterson A Addington J Negative symptom interventions in youth at risk of psychosis: a systematic review and network meta-analysis. Schizophr Bull (2018) 44:807–23. 10.1093/schbul/sbx139 PMC6007754 29069511

[63] Hutton P Taylor PJ Cognitive behavioural therapy for psychosis prevention: a systematic review and meta-analysis. Psychol Med (2014) 44:449–68. 10.1017/S0033291713000354 23521867

[64] Devoe DJ Farris MS Townes P Addington J Interventions and social functioning in youth at risk of psychosis: a systematic review and meta-analysis. Early Interv Psychiatry (2018) 13(2):169–180. 10.1111/eip.12689 29938910

[65] Ising HK Kraan TC Rietdijk J Dragt S Klaassen RMC Boonstra N Four-year follow-up of cognitive behavioral therapy in persons at ultra-high risk for developing psychosis: the dutch early detection intervention evaluation (EDIE-NL) Trial. Schizophr Bull (2016) 42:1243–52. 10.1093/schbul/sbw018 PMC4988735 26994397

[66] McGorry PD Nelson B Phillips LJ Yuen HP Francey SM Thampi A Randomized controlled trial of interventions for young people at ultra-high risk of psychosis: twelve-month outcome. J Clin Psychiatry (2013) 74:349–56. 10.4088/JCP.12m07785 23218022

[67] Radua J Ramella-Cravaro V Ioannidis JPA Reichenberg A Phiphopthatsanee N Amir T What causes psychosis? An umbrella review of risk and protective factors. World Psychiatry (2018) 17:49–66. 29352556 10.1002/wps.20490 PMC5775150

[68] Addington J Epstein I Liu L French P Boydell KM Zipursky RB A randomized controlled trial of cognitive behavioral therapy for individuals at clinical high risk of psychosis. Schizophrenia Res (2011) 125:54–61. 10.1016/j.schres.2010.10.015 21074974

[69] Bechdolf A Müller H Stützer H Lambert M Karow A Zink M 108. PREVENT: a Randomized Controlled Trial for the Prevention of First-Episode Psychosis Comparing Cognitive–Behavior Therapy (CBT), Clinical Management and Aripiprazole Combined and Clinical Management and Placebo Combined. Schizophr Bull (2016) 43:S56. 10.1093/schbul/sbx021.146

[70] Cadenhead K Addington J Cannon T Cornblatt B Mathalon D McGlashan T Omega-3 Fatty Acid Versus Placebo in a Clinical High-Risk Sample From the North American Prodrome Longitudinal Studies (NAPLS) Consortium. Schizophr Bull (2017) 43:S16–6. 10.1093/schbul/sbx021.04223.

[71] Choi J Corcoran CM Fiszdon JM Stevens M Javitt DC Deasy M Pupillometer-based neurofeedback cognitive training to improve processing speed and social functioning in individuals at clinical high risk for psychosis. Psychiatr Rehabil J (2017) 40:33–42. 10.1037/prj0000217 27560455 PMC5326611

[72] Kantrowitz JT Woods SW Petkova E Cornblatt B Corcoran CM Chen H D-serine for the treatment of negative symptoms in individuals at clinical high risk of schizophrenia: a pilot, double-blind, placebo-controlled, randomised parallel group mechanistic proof-of-concept trial. Lancet Psychiatry (2015) 2:403–12. 10.1016/S2215-0366(15)00098-X 26360284

[73] Loewy R Fisher M Schlosser DA Biagianti B Stuart B Mathalon DH Intensive auditory cognitive training improves verbal memory in adolescents and young adults at clinical high risk for psychosis. Schizophr Bull (2016) 42 suppl 1:s118–26. 10.1093/schbul/sbw009 PMC4960436 26903238

[74] Miklowitz DJ O’Brien MP Schlosser DA Addington J Candan KA Marshall C Family-focused treatment for adolescents and young adults at high risk for psychosis: results of a randomized trial. J Am Acad Child Adolesc Psychiatry (2014) 53:848–58. 10.1016/j.jaac.2014.04.020 PMC4112074 25062592

[75] Piskulic D Barbato M Liu L Addington J Pilot study of cognitive remediation therapy on cognition in young people at clinical high risk of psychosis. Psychiatry Res (2015) 225:93–8. 10.1016/j.psychres.2014.10.021 25467705

[76] Ruhrmann S Bechdolf A Kuhn KU Wagner M Schultze-Lutter F Janssen B Acute effects of treatment for prodromal symptoms for people putatively in a late initial prodromal state of psychosis. Br J Psychiatry (2007) Suppl 51:s88–95. 10.1192/bjp.191.51.s88 18055944

[77] Stain HJ Bucci S Baker AL Carr V Emsley R Halpin S A randomised controlled trial of cognitive behaviour therapy versus non-directive reflective listening for young people at ultra high risk of developing psychosis: The detection and evaluation of psychological therapy (DEPTh) trial. Schizophrenia Res (2016) 176:212–9. 10.1016/j.schres.2016.08.008 27554197

[78] van der Gaag M Nieman DH Rietdijk J Dragt S Ising HK Klaassen RMC Cognitive behavioral therapy for subjects at ultrahigh risk for developing psychosis: a randomized controlled clinical trial. Schizophr Bull (2012) 38:1180–8. 10.1093/schbul/sbs105 PMC3494039 22941746

[79] Woods SW Ziprasidone in the Psychosis Prodrome (ZIP) ClinicalTrials.gov Identifier: NCT00635700 [Last updated: September 6, 2016]. ClinicalTrials.gov (2016).

[80] Woods S Saksa J Compton M Daley M Rajarethinam R Graham K 112. Effects of Ziprasidone Versus Placebo in Patients at Clinical High Risk for Psychosis. Schizophr Bull (2017) 43:S58–8. 10.1093/schbul/sbx021.150

[81] Yung AR Phillips LJ Nelson B Francey SM PanYuen H Simmons MB Randomized controlled trial of interventions for young people at ultra high risk for psychosis. J Clin Psychiatry (2011) 72:430–40. 10.4088/JCP.08m04979ora 21034687

[82] Green MF Penn DL Bentall R Carpenter WT Gaebel W Gur RC Social cognition in schizophrenia: an NIMH workshop on definitions assessment, and research opportunities. Schizophr Bull (2008) 34:1211–20. 10.1093/schbul/sbm145 PMC2632490 18184635

[83] Fett AK Viechtbauer W Dominguez MD Penn DL van Os J Krabbendam L The relationship between neurocognition and social cognition with functional outcomes in schizophrenia: a meta-analysis. Neurosci Biobehav Rev (2011) 35:573–88. 10.1016/j.neubiorev.2010.07.001 20620163

[84] Fusar-Poli P Deste G Smieskova R Barlati S Yung AR Howes O Cognitive functioning in prodromal psychosis: a meta-analysis. Arch Gen Psychiatry (2012) 69:562–71. 10.1001/archgenpsychiatry.2011.1592 22664547

[85] van Donkersgoed RJ Wunderink L Nieboer R Aleman A Pijnenborg GH Social cognition in individuals at ultra-high risk for psychosis: a meta-analysis. PLoS One (2015) 10:e0141075. 10.1371/journal.pone.0141075 26510175 PMC4624797

[86] Feifel D Reza T Oxytocin modulates psychotomimetic-induced deficits in sensorimotor gating. Psychopharmacology (Berl) (1999) 141:93–8. 10.1007/s002130050811 9952070

[87] Lee PR Brady DL Shapiro RA Dorsa DM Koenig JI Social interaction deficits caused by chronic phencyclidine administration are reversed by oxytocin. Neuropsychopharmacology (2005) 30:1883–94. 10.1038/sj.npp.1300722 15798779

[88] Kosfeld M Heinrichs M Zak PJ Fischbacher U Fehr E Oxytocin increases trust in humans. Nature (2005) 435:673–6. 10.1038/nature03701 15931222

[89] Eckstein M Becker B Scheele D Scholz C Preckel K Schlaepfer TE Oxytocin facilitates the extinction of conditioned fear in humans. Biol Psychiatry (2015) 78:194–202. 10.1016/j.biopsych.2014.10.015 25542304

[90] Zink CF Meyer-Lindenberg A Human neuroimaging of oxytocin and vasopressin in social cognition. Horm Behav (2012) 61:400–9. 10.1016/j.yhbeh.2012.01.016 PMC3312952 22326707

[91] Pedersen CA Gibson CM Rau SW Salimi K Smedley KL Casey RL Intranasal oxytocin reduces psychotic symptoms and improves Theory of Mind and social perception in schizophrenia. Schizophr Res (2011) 132:50–3. 10.1016/j.schres.2011.07.027 21840177

[92] Hofmann SG Fang A Brager DN Removal notice to Effect of Intranasal Oxytocin Administration on Psychiatric Symptoms: A Meta-Analysis of Placebo-Controlled Studies Psychiatr Res. Psychiatry Res (2015) 228263708–142018) 299. 10.1016/j.psychres.2018.04.028 PMC6208317 29678249

[93] Williams DR Burkner PC Effects of intranasal oxytocin on symptoms of schizophrenia: a multivariate Bayesian meta-analysis. Psychoneuroendocrinology (2017) 75:141–51. 10.1016/j.psyneuen.2016.10.013 27825069

[94] Bethlehem RA van Honk J Auyeung B Baron-Cohen S Oxytocin brain physiology, and functional connectivity: a review of intranasal oxytocin fMRI studies. Psychoneuroendocrinology (2013) 38:962–74. 10.1016/j.psyneuen.2012.10.011 23159011

[95] Grace SA Rossell SL Heinrichs M Kordsachia C Labuschagne I Oxytocin and brain activity in humans: a systematic review and coordinate-based meta-analysis of functional MRI studies. Psychoneuroendocrinology (2018) 96:6–24. 10.1016/j.psyneuen.2018.05.031 29879563

[96] Paloyelis Y Doyle OM Zelaya FO Maltezos S Williams SC Fotopoulou A A spatiotemporal profile of In Vivo cerebral blood flow changes following intranasal oxytocin in humans. Biol Psychiatry (2016) 79:693–705. 10.1016/j.biopsych.2014.10.005 25499958

[97] Davies C Paloyelis Y Rutigliano G Cappucciati M De Micheli A Ramella-Cravaro V Oxytocin modulates hippocampal perfusion in people at clinical high risk for psychosis. Neuropsychopharmacology (2019) 44(7):1300–1309. 10.1038/s41386-018-0311-6 30626906 PMC6784972

[98] McGuire P Robson P Cubala WJ Vasile D Morrison PD Barron R Cannabidiol (CBD) as an adjunctive therapy in schizophrenia: a multicenter randomized controlled trial. Am J Psychiatry (2018) 175:225–31. 10.1176/appi.ajp.2017.17030325 29241357

[99] Leweke FM Piomelli D Pahlisch F Muhl D Gerth CW Hoyer C Cannabidiol enhances anandamide signaling and alleviates psychotic symptoms of schizophrenia. Transl Psychiatry (2012) 2:e94. 10.1038/tp.2012.15 22832859 PMC3316151

[100] Bhattacharyya S Wilson R Appiah-Kusi E O'Neill A Brammer M Perez J Effect of cannabidiol on medial temporal, midbrain and striatal dysfunction in people at clinical high risk of psychosis: a randomized clinical trial. JAMA Psychiatry (2018) 75:1107–17. 10.1001/jamapsychiatry.2018.2309 PMC6248101 30167644

[101] Miladinovic B Hozo I Djulbegovic B Trial sequential boundaries for cumulative meta-analyses. Stata J (2013) 13:77–91. 10.1177/1536867X1301300106

[102] Fusar-Poli P Rutigliano G Stahl D Schmidt A Ramella-Cravaro V Shetty H Deconstructing pretest risk enrichment to optimize prediction of psychosis in individuals at clinical high risk. Jama Psychiatry (2016) 73:1260–7. 10.1001/jamapsychiatry.2016.2707 27784037

[103] Fusar-Poli P Rutigliano G Stahl D Davies C Bonoldi I Reilly T Development and validation of a clinically based risk calculator for the transdiagnostic prediction of psychosis. Jama Psychiatry (2017) 74:493–500. 10.1001/jamapsychiatry.2017.0284 28355424 PMC5470394

[104] McGorry PD Hartmann JA Spooner R Nelson B Beyond the "at risk mental state" concept: transitioning to transdiagnostic psychiatry. World Psychiatry (2018) 17:133–42. 10.1002/wps.20514 PMC5980504 29856558

[105] Fusar-Poli P Oliver D Spada G Patel R Stewart R Dobson R Real-world Implementation of a Transdiagnostic risk calculator for the automatic detection of individuals at risk of psychosis in clinical routine: study protocol. Front Psychiatry (2019) 10:109. 10.3389/fpsyt.2019.00109 30949070 PMC6436079

[106] McDonald M Christoforidou E Van Rijsbergen N Gajwani R Gross J Gumley AI Using online screening in the general population to detect participants at clinical high-risk for psychosis. Schizophr Bull (2019) 45(3):600–609. 10.1093/schbul/sby069 29889271 PMC6483579

[107] Millan MJ Andrieux A Bartzokis G Cadenhead K Dazzan P Fusar-Poli P Altering the course of schizophrenia: progress and perspectives. Nat Rev Drug Discov (2016) 5:485–51. 10.1038/nrd.2016.28 26939910

[108] Bosnjak Kuharic D Kekin I Hew J Rojnic Kuzman M Puljak L Interventions for prodromal stage of psychosis. Cochrane Database Syst Rev 2019;2019(11). 10.1002/14651858.CD012236.pub2 PMC6823626 31689359

[109] Davies C Cipriani A Radua J Provenzani U McGuire P Fusar-Poli P Preventative treatments for psychosis: a network meta-analysis protocol. PROSPERO Int Prospective Register Syst Rev (2017).

[110] Hartmann JA McGorry PD Schmidt SJ Amminger GP Yuen HP Markulev C Opening the black box of cognitive-behavioural case management in clients with ultra-high risk for psychosis. Psychother Psychosom (2017) 3052:292–9. 10.1159/000477551 28903120

[111] Cipriani A Higgins JPT Geddes JR Salanti G Conceptual and technical challenges in network meta-analysis. Ann Internal Med (2013) 159:130–7. 10.7326/0003-4819-159-2-201307160-00008 23856683

[112] Thorlund K Mills EJ Sample size and power considerations in network meta-analysis. Syst Rev (2012) 1:41. 10.1186/2046-4053-1-41 22992327 PMC3514119

[113] Kanters S Ford N Druyts E Thorlund K Mills EJ Bansback N Use of network meta-analysis in clinical guidelines. Bull World Health Org (2016) 94:782–4. 10.2471/BLT.16.174326 PMC5043215 27843171

[114] Leucht S Chaimani A Cipriani AS Davis JM Furukawa TA Salanti G Network meta-analyses should be the highest level of evidence in treatment guidelines. Eur Arch Psychiatry Clin Neurosci (2016) 266:477–80. 10.1007/s00406-016-0715-4 27435721

[115] Fusar-Poli P Van Os J Lost in transition: setting the psychosis threshold in prodromal research. Acta Psychiatr Scand (2013) 127:248–52. 10.1111/acps.12028 23136851

[116] Fusar-Poli P Cappucciati M De Micheli A Rutigliano G Bonoldi I Tognin S Diagnostic and prognostic significance of brief limited intermittent psychotic symptoms (BLIPS) in individuals at ultra high risk. Schizophr Bull (2017) 43:48–56. 10.1093/schbul/sbw151 28053130 PMC5216865

[117] Fusar-Poli P Cappucciati M Bonoldi I Hui LM Rutigliano G Stahl DR Prognosis of brief psychotic episodes: a meta-analysis. JAMA Psychiatry (2016) 73:211–20. 10.1001/jamapsychiatry.2015.2313 26764163

[118] Moll van Charante EP Richard E Eurelings LS van Dalen JW Ligthart SA van Bussel EF Effectiveness of a 6-year multidomain vascular care intervention to prevent dementia (preDIVA): a cluster-randomised controlled trial. Lancet (2016) 388:797–805. 10.1016/S0140-6736(16)30950-3 27474376

[119] Frankish H Horton R Prevention and management of dementia: a priority for public health. Lancet (2017) 390:2614–5. 10.1016/S0140-6736(17)31756-7 28735854

[120] Oliver D Davies C Crossland G Lim S Gifford G McGuire P Can we reduce the duration of untreated psychosis? A systematic review and meta-analysis of controlled interventional studies. Schizophr Bull (2018) 44(6):1362–1372. 10.1093/schbul/sbx166 29373755 PMC6192469

[121] Altman DG Bland JM Absence of evidence is not evidence of absence. BMJ (1995) 311(7003):485. 10.1136/bmj.311.7003.485 7647644 PMC2550545

[122] Cuzick J Preventive therapy for cancer. Lancet Oncol (2017) 18:e472–82. 10.1016/S1470-2045(17)30536-3 28759386

[123] Fusar-Poli P Schultze-Lutter F Predicting the onset of psychosis in patients at clinical high risk: practical guide to probabilistic prognostic reasoning. Evid Based Ment Health (2016) 19:10–5. 10.1136/eb-2015-102295 PMC10699348 26792832

[124] Fusar-Poli P Why ultra high risk criteria for psychosis prediction do not work well outside clinical samples and what to do about it. World Psychiatry (2017) 16:212–3. 10.1002/wps.20405 PMC5428173 28498578

[125] Fusar-Poli P Schultze-Lutter F Addington J Intensive community outreach for those at ultra high risk of psychosis: dilution not solution. Lancet Psychiatry (2016) 3:18. 10.1016/S2215-0366(15)00491-5 26772061

[126] Fusar-Poli P Bonoldi I Yung AR Borgwardt S Kempton MJ Valmaggia L Predicting psychosis: meta-analysis of transition outcomes in individuals at high clinical risk. Arch Gen Psychiatry (2012) 69:220–9. 10.1001/archgenpsychiatry.2011.1472 22393215

[127] Fusar-Poli P Palombini E Davies C Oliver D Bonoldi I Ramella-Cravaro V Why transition risk to psychosis is not declining at the OASIS ultra high risk service: The hidden role of stable pretest risk enrichment. Schizophr Res (2017) 10.1016/j.schres.2017.06.015 28734908

[128] Schmidt A Cappucciati M Radua J Rutigliano G Rocchetti M Dell'Osso L Improving prognostic accuracy in subjects at clinical high risk for psychosis: systematic review of predictive models and meta-analytical sequential testing simulation. Schizophr Bull (2017) 43:375–88. 10.1093/schbul/sbw098 PMC5605272 27535081

[129] Kane JM Correll CU omega-3 Polyunsaturated Fatty Acids to Prevent Psychosis: the Importance of Replication Studies. JAMA Psychiatry (2017) 74:11–2. 10.1001/jamapsychiatry.2016.2945 27893036

[130] Rutigliano G Merlino S Minichino A Patel R Davies C Oliver D Long term outcomes of acute and transient psychotic disorders: The missed opportunity of preventive interventions. Eur Psychiatry (2018) 52:126–33. 10.1016/j.eurpsy.2018.05.004 29787962

[131] Minichino A Rutigliano G Merlino S Davies C Oliver D De Micheli A Unmet needs in patients with brief psychotic disorders: too ill for clinical high risk services and not enough ill for first episode services. Eur Psychiatry (2019) 57:26–32. 10.1016/j.eurpsy.2018.12.006 30658277

[132] Cannon TD Yu C Addington J Bearden CE Cadenhead KS Cornblatt BA An Individualized Risk Calculator for Research in Prodromal Psychosis. Am J Psychiatry (2016) 173(10):980–988. 10.1176/appi.ajp.2016.15070890 27363508 PMC5048498

[133] Koutsouleris N Kambeitz-Ilankovic L Ruhrmann S Rosen M Ruef A Dwyer DB Prediction Models of functional outcomes for individuals in the clinical high-risk state for psychosis or with recent-onset depression: a multimodal, multisite machine learning analysis. JAMA Psychiatry (2018) 75:1156–72. 10.1001/jamapsychiatry.2018.2165 PMC6248111 30267047

[134] Uher R Zwicker A Etiology in psychiatry: embracing the reality of poly-gene-environmental causation of mental illness. World Psychiatry (2017) 16:121–9. 10.1002/wps.20436 PMC5428165 28498595

[135] Thomas J Noel-Storr A Marshall I Wallace B McDonald S Mavergames C Living systematic reviews: 2. Combining human and machine effort. J Clin Epidemiol (2017) 91:31–7. 10.1016/j.jclinepi.2017.08.011 28912003

[136] O'Brien D Harvey K Howse J Reardon T Creswell C Barriers to managing child and adolescent mental health problems: a systematic review of primary care practitioners' perceptions. Br J Gen Pract (2016) 66:e693–707. 10.3399/bjgp16X687061 27621291 PMC5033306

[137] HM Government No health without mental health: implementation framework. in: https://www.gov.uk/government/uploads/,system/uploads/attachment_data/file/215811/, and d.p.a.S. 2016). (Eds.), (2011).

[138] NHS England Future in mind: promoting protecting and improving our children and young people’s mental health and wellbeing. Department of Health (2015).

[139] Davey CG McGorry PD Early intervention for depression in young people: a blind spot in mental health care. Lancet Psychiatry (2019) 6(3):267–272. 10.1016/S2215-0366(18)30292-X 30502077

[140] Vieta E Salagre E Grande I Carvalho AF Fernandes BS Berk M Early intervention in bipolar disorder. Am J Psychiatry (2018) 175:411–26. 10.1176/appi.ajp.2017.17090972 29361850

[141] Fusar-Poli P De Micheli A Rocchetti M Cappucciati M Ramella-Cravaro V Rutigliano G Semistructured Interview for Bipolar At Risk States (SIBARS). Psychiatry Res (2018) 264:302–9. 10.1016/j.psychres.2018.03.074 29665559

[142] McGorry PD Mei C Early intervention in youth mental health: progress and future directions. Evid Based Ment Health (2018) 21:182–4. 10.1136/ebmental-2018-300060 PMC10270418 30352884

[143] McGorry PD Goldstone SD Parker AG Rickwood DJ Hickie IB Cultures for mental health care of young people: an Australian blueprint for reform. Lancet Psychiatry (2014) 1:559–68. 10.1016/S2215-0366(14)00082-0 26361315

[144] Hetrick SE Bailey AP Smith KE Malla A Mathias S Singh SP Integrated (one-stop shop) youth health care: best available evidence and future directions. Med J Aust (2017) 207:S5–S18. 10.5694/mja17.00694 29129182

[145] Hagan CC Graham JM Wilkinson PO Midgley N Suckling J Sahakian BJ Neurodevelopment and ages of onset in depressive disorders. Lancet Psychiatry (2015) 2:1112–6. 10.1016/S2215-0366(15)00362-4 26613851

[146] ClinicalTrials.gov The Staged Treatment in Early Psychosis Study (STEP). U.S. National Library of Medicine (2019).

