# Supplementary material for: Preventive Treatments for Psychosis: Umbrella Review (Just the Evidence)
Source: Front Psychiatry. 2019 Dec 11;10:764. doi: 10.3389/fpsyt.2019.00764 (PMC6917652; doi:10.3389/fpsyt.2019.00764)
Supplement: Supplementary file 1 [file DataSheet_1.docx]

**Figure S1.** Forest plot for the Network Meta-Analysis of randomised controlled trials testing the impact of preventive treatments on the onset of psychosis in CHR-P individuals. The original analyses are fully detailed in Davies et al 2018[30]. Needs-based interventions (NBI); Cognitive Behavioural Therapy, French & Morrison protocol (CBT-F); Cognitive Behavioural Therapy, van der Gaag protocol (CBT-V); D-serine (Dser); integrated psychological interventions (IPI); aripiprazole (ARI); olanzapine (OLA); ziprasidone (ZIP); family therapy (FFT).

*6-month outcome*

*12-month outcome*

**Table S1**

| **Sampling** | **Recruitment (pretest)** | **Psychometric assessment (post-test)** | | **Total sample size excluding attrition ^(c)^** | | |
| --- | --- | --- | --- | --- | --- | --- |
| Type of sample | Risk of psychosis at 3yrs | Risk of psychosis at 3yrs | | Risks Ratio (Risk experimental treatment / Risk needs-based intervention | | |
|  |  | *CHR+ ^(a)^* | *CHR- ^(b)^* | *0.5* | *0.6* | *0.7* |
| Pretest risk in people undergoing CHR assessment outside randomised clinical trials | 0.03^(d)^-0.49 | 0.051 | 0.003 | 1770 | 2946 | 5556 |
| General population | 0.004 | 0.007 | <0.001 | 13378 | 22292 | 42100 |
| Neurapro trial -control arm | 0.082 | 0.140 | 0.008 | 596 | 988 | 1860 |
| Pretest risk stratification |  |  |  |  |  |  |
| *low pretest risk* | 0.014 | 0.026 | 0.001 | 3534 | 5886 | 11110 |
| *moderately low pretest risk* | 0.100 | 0.168 | 0.010 | 484 | 804 | 1508 |
| *moderately high pretest risk* | 0.181 | 0.287 | 0.020 | 250 | 414 | 774 |
| *high pretest risk* | 0.456 | 0.604 | 0.070 | 78 | 128 | 232 |
| (a) LR+ = 1.82; (b) LR- = 0.09; (c) alpha=0.05; power 80%; 2-sided; allocation ratio = 1. Post-test probability=LR*pretest probability/[(1-pretest probability)+(pretest probability*LR)]. The sample sizes reported in the table indicate the individuals who should complete the trial (to estimate the baseline sample, attrition should be considered); (d) the average is 0.15 but because it depends on unstandardised idiosyncratic recruitment strategies, it is highly variable ranging from 3% to 49% depending on the study[5]. It tends to be on the lower side when the recruitment focuses on children and adolescent populations, intermediate when the recruitment focuses on primary care settings and higher when the recruitment focuses on secondary care. In this table to allow a conservative estimate of the sample size required we use only the lower bound of 0.03. | | | | | | |
